# Digital Health Technologies for Mobility Recovery Among Intensive Care Unit Survivors: Scoping Review

**DOI:** 10.2196/105445

**Published:** 2026-11-05

**Authors:** Yuhang Yan, Jiayan Zhu, Sining Peng, Jin Qiu, Linghong Cheng, Xiaohua Ge

**Affiliations:** 1 School of Nursing, Shanghai Jiao Tong University, Shanghai, China; 2 Department of Anesthesiology and Surgical Intensive Care Unit, Xinhua Hospital, Shanghai, China; 3 Department of Health Humanities Education, Xinhua Hospital Affiliated to Shanghai Jiao Tong University School of Medicine, No. 1665 Kongjiang Road, Yangpu District, Shanghai, 200082, China, 86 13651633618

**Keywords:** digital health, intensive care units, survivors, critical illness, rehabilitation, telemedicine, mobility limitation

## Abstract

**Background:**

Mobility recovery after critical illness is a complex and multidimensional process. Digital health technologies have emerged as approaches for delivering rehabilitation, monitoring recovery, and facilitating self-management among intensive care unit (ICU) survivors. However, the extent to which current digital health approaches address the multidimensional nature of mobility recovery remains unclear.

**Objective:**

This scoping review aimed to map the current evidence on digital health approaches supporting mobility recovery among ICU survivors and to examine the mobility domains, outcomes, feasibility, and acceptability associated with these approaches.

**Methods:**

A scoping review was conducted following the Arksey and O’Malley framework and was reported according to the Preferred Reporting Items for Systematic Reviews and Meta-Analyses Extension for Scoping Reviews (PRISMA-ScR). PubMed, Embase, CINAHL, Web of Science, and the Cochrane Library were searched for studies published from January 2016 to September 21, 2026. Studies involving adult ICU survivors that used digital health approaches to assess, monitor, support, or promote mobility recovery were included. Mobility outcomes were mapped to the International Classification of Functioning, Disability and Health domains of body functions and structures, activities, and participation. Clinical outcomes were synthesized separately from implementation and user experience outcomes.

**Results:**

Twenty-seven reports representing 24 unique parent studies were included. Digital health approaches comprised telemedicine, telerehabilitation, mobile health, wearable monitoring, virtual reality, robot-assisted rehabilitation, and digital recovery platforms. Telemedicine and telerehabilitation were the most frequently reported approaches, and exercise-based rehabilitation was the predominant intervention component. Mobility outcomes were heterogeneous and concentrated on body functions and structures and activity-level performance, particularly muscle strength, exercise capacity, and functional performance, whereas participation outcomes were less frequently represented. Controlled and comparative studies showed mixed between-group findings, with clinical benefits not consistently demonstrated across primary or mobility-related outcomes. In contrast, feasibility, adherence, usability, and acceptability were generally favorable when assessed, although technical difficulties and incomplete device-generated data were reported in some studies.

**Conclusions:**

Digital health technologies offer diverse approaches to supporting mobility recovery after critical illness, but current evidence remains concentrated on physical function and activity-level outcomes, with limited attention to participation and long-term recovery. More rigorous and longitudinal studies using standardized, multidimensional mobility outcomes are needed to clarify clinical effectiveness and support sustainable implementation.

## Introduction

Critical care has entered a survivorship era. Improvements in intensive care unit (ICU) survival have resulted in a growing population of ICU survivors experiencing post–intensive care syndrome, including physical, cognitive, psychological, and social impairments [1-3]. Among these domains, mobility is clinically central, as it determines whether survivors can safely transfer, walk indoors and outdoors, climb stairs, engage in rehabilitation activities, return to work or caregiving roles, and re-engage in meaningful daily activities [4,5]. Longitudinal studies among survivors of acute respiratory distress syndrome, sepsis, and other critical illnesses have consistently demonstrated persistent physical disability, reduced walking capacity, dependence in activities of daily living (ADLs), frailty, and poorer quality of life [6-8].

Mobility in ICU survivorship extends beyond ambulation and encompasses multiple dimensions of functioning [9-13]. The World Health Organization’s International Classification of Functioning, Disability and Health (ICF) provides a standardized framework for describing functioning across body functions and structures, activities, and participation, while recognizing the influence of contextual factors [14]. Accordingly, mobility recovery after critical illness can be examined across these complementary domains. ICU survivor studies have used diverse measures, including the 6-minute walk test (6MWT), functional status score for the ICU [9], de Morton Mobility Index [15], short physical performance battery [16], Barthel Index [17] or Katz ADL [18], frailty instruments [19], handgrip or knee-extension dynamometry, ultrasound muscle thickness [20], 36-Item Short Form Health Survey (SF-36) [21], and qualitative accounts of meaningful activity.

Despite this multidimensional conceptualization, it remains unclear whether existing digital health technologies address mobility recovery across the ICF domains or continue to focus predominantly on physical capacity and performance. Identifying which domains are represented in current digital health research is, therefore, important for revealing evidence gaps and informing more comprehensive, person-centered rehabilitation strategies for ICU survivors.

Clinic-based assessments provide standardized snapshots, but they may not fully capture fluctuations in mobility and participation in everyday settings. Many ICU survivors experience fatigue, dyspnea, weakness, mood symptoms, transportation barriers, and fragmented services after discharge [22-24]. A survivor may perform adequately on a 6MWT in clinic yet remain sedentary at home; another may accumulate daily steps but continue to avoid stairs, community mobility, or social participation. These limitations highlight the need for approaches that can support and monitor recovery continuously in real-world settings.

Digital health technologies offer an opportunity. By leveraging smartphones, wearable devices, tele–video sessions, app-based exercise programs, virtual reality (VR), and structured digital follow-up pathways [25-29], these interventions can deliver remote rehabilitation, individualized exercise guidance, real-time feedback, motivational support, and structured progression [25,27,30-34]. However, the existing evidence remains fragmented, with many studies being small-scale, feasibility-oriented, and primarily focused on short-term physical outcomes.

A clear synthesis is therefore needed to determine which digital health technologies have been applied to mobility recovery among ICU survivors and which dimensions of functioning they address. Accordingly, this scoping review focuses on adult ICU survivors, encompasses digital health modalities across the post-ICU recovery trajectory, maps mobility outcomes to the ICF domains of body functions and structures, activities, and participation, and separately examines clinical outcomes, implementation feasibility, and user experience.

## Methods

### Study Design

This scoping review was conducted in accordance with the methodological framework proposed by Arksey and O’Malley [35], following their 5-stage approach. The review was reported according to the PRISMA-ScR (Preferred Reporting Items for Systematic Reviews and Meta-Analyses Extension for Scoping Reviews) guidelines [36]. The completed PRISMA-ScR checklist is provided in Checklist 1. The study protocol and analysis plan were prospectively registered on the Open Science Framework [37].

### Formulating the Research Question

This scoping review aimed to systematically map the current evidence on digital health technologies to support mobility recovery among ICU survivors. The review addressed the following research questions: (1) What digital health technologies have been applied to support mobility recovery among ICU survivors? (2) Which dimensions of mobility are targeted by these digital health technologies? (3) What evidence exists regarding mobility-related outcomes, implementation experiences, and user perspectives associated with these digital health technologies?

### Eligibility Criteria

The eligibility criteria were developed according to the PCC (population, concept, and context) framework [38].

#### Inclusion Criteria

The inclusion criteria for the study are as follows:

1. Population: adults aged 18 years or older who survived critical illness following an ICU admission.
2. Concept: studies describing, evaluating, or implementing digital health technologies, programs, services, platforms, or interventions designed to support, assess, monitor, or promote mobility recovery after critical illness. Mobility was operationalized according to the ICF framework as outcomes related to body functions and structures, activities, and participation. Self-management and related behavioral or educational components were considered intervention or implementation characteristics rather than mobility domains.
3. Context: studies conducted across the ICU recovery trajectory, including transitions from hospital to home, outpatient follow-up, and community-based rehabilitation settings.

#### Exclusion Criteria

Studies were excluded if the digital health technology was not relevant to mobility or functional recovery, if neither mobility-related nor relevant implementation or user experience outcomes were reported, or if the study did not include an identifiable group of adult ICU survivors. Nonempirical publications, including reviews, editorials, commentaries, conceptual papers, and study protocols, were also excluded. The full eligibility criteria are presented in Table 1.

**Table 1.** Eligibility criteria based on the population-concept-context framework.

Domain	Inclusion	Exclusion
Population	Adults (≥18 y) who survived critical illness following an ICU^a^ admission, including individuals transitioning from the ICU to hospital wards and those undergoing postdischarge recovery; studies of mixed populations were eligible if adult ICU survivors could be identified within the study population	Pediatric or neonatal populations; studies that did not include an identifiable group of adult ICU survivors
Concept	Digital health technologies, programs, services, platforms, or interventions that explicitly supported, assessed, monitored, or promoted mobility-related recovery or physical rehabilitation in populations including adult ICU survivors; eligible technologies included mobile apps, websites, wearable devices, telephones, video consultations, virtual reality, digital recovery platforms, and other digitally enabled approaches	Digital technologies used solely for assessment or monitoring without a clear relevance to mobility recovery; interventions delivered exclusively during ICU admission; nondigital rehabilitation technologies
Context	Any setting across the ICU recovery trajectory, including hospital wards, outpatient clinics, home-based settings, and community rehabilitation following ICU discharge	Settings unrelated to recovery after critical illness; pediatric or neonatal ICUs
Outcomes	Mobility-related outcomes included body functions and structures, activities, and participation; implementation and user experience outcomes included feasibility, adherence, usability, acceptability, satisfaction, self-management support, self-efficacy, engagement, and perceived barriers or facilitators	Studies reporting neither mobility-related outcomes nor relevant implementation or user experience outcomes; studies reporting only mortality or unrelated outcomes
Study design	All primary empirical study designs, including randomized controlled trials, nonrandomized studies, feasibility and pilot studies, observational studies, mixed methods studies, qualitative studies, case reports, and empirical innovation reports	Study protocols, reviews, editorials, conference abstracts, commentaries, conceptual papers, and other nonempirical publications
Publication	Peer-reviewed full-text papers published in English from January 2016 onward	Publications before January 2016, non-English publications, or papers without full-text availability

a ICU: intensive care unit.

### Search Strategy

#### Information Sources, Search Dates, and Limits

We systematically searched PubMed, Embase, CINAHL, Web of Science, and the Cochrane Library for records published from January 1, 2016, through June 2026. To capture newly published studies, the searches were updated in all 5 databases on September 21, 2026, which was the final search date. No language, study design, or publication-type filters were applied during the database searches; all eligibility restrictions were applied during study selection.

#### Search Terms and Search Combinations

The search strategy was developed in consultation with a medical librarian and informed by the population, concept, and context framework. Controlled vocabulary and free-text terms were used to represent three concepts: (1) ICU survivors and critical illness, (2) digital health technologies, and (3) mobility-related recovery. Synonymous terms within each concept were combined using OR, and the 3 concept blocks were combined using AND. The NOT operator was not used. Quotation marks were used for multiword phrases, and truncation symbols were applied where supported by the database.

The complete database-specific search strategies, including controlled vocabulary, keywords, field tags, Boolean operators, phrase searching, truncation, date limits, final search dates, and numbers of records retrieved, are provided in Multimedia Appendix 1.

#### Study Selection

All retrieved records were imported into EndNote for the removal of duplicates. Records identified through the updated searches were added to the same reference library and deduplicated against records retrieved previously. Two reviewers independently screened titles and abstracts against predefined eligibility criteria and subsequently assessed potentially eligible full-text papers. Disagreements were resolved through discussion or consultation with a third reviewer. The updated study-selection process, including records identified through the final search update, was documented using a PRISMA flow diagram.

#### Data Charting

Data were independently charted by 2 reviewers (YY and JZ) using a standardized Microsoft Excel form. Discrepancies were resolved through discussion or consultation with a third reviewer (SP). The charted information included author, publication year, country, study design, participant characteristics, digital health technology, intervention characteristics, mobility-related outcomes, statistical or analytical approach, reported procedures for handling home-generated digital or device data, where applicable, and principal findings.

Mobility-related outcomes were classified according to the ICF framework [14] into body functions and structures, activities, and participation. Outcomes were coded at the outcome-measure level; therefore, a study could contribute to more than one ICF domain when distinct outcomes represented different domains. Individual outcome measures were assigned to the domain they most directly represented and were not double-counted across domains. Self-management, goal setting, education, symptom monitoring, and engagement were treated as intervention- or implementation-related components rather than mobility domains.

For studies with a comparator, details of the comparator, primary and secondary outcomes as defined by the original study authors, assessment time points, between-group findings, and within-group changes were extracted where available. Implementation and user-experience outcomes, including feasibility, retention, adherence, intervention completion, safety, usability, acceptability, satisfaction, and perceived barriers or facilitators, were charted separately from clinical mobility outcomes.

Theoretical frameworks were extracted only when a named theory, model, or framework was explicitly reported as informing intervention design, implementation, or analysis. Frameworks were recorded as described by the original authors and were not inferred when no explicit theoretical basis was reported.

#### Data Synthesis

Because of heterogeneity in study designs, interventions, and outcome measures, data were synthesized descriptively. Digital health technologies were classified according to their predominant function and delivery mode into 7 categories: telemedicine, telerehabilitation, mobile health, wearable monitoring, VR, robot-assisted rehabilitation, and digital recovery platforms. Each report was assigned to one primary technology category based on the predominant digital delivery mechanism; multicomponent interventions were classified according to the component through which the principal rehabilitation or recovery support was delivered.

Mobility-related outcomes were synthesized according to the ICF domains of body functions and structures, activities, and participation. A report could contribute to more than one ICF domain when it included distinct outcome measures representing different domains. Clinical outcomes were synthesized separately from implementation and user experience outcomes. In comparative studies, primary outcomes and between-group findings were prioritized; within-group changes were not interpreted as evidence of intervention effectiveness in the absence of a corresponding between-group effect. In noncomparative studies, changes over time were described without inferring effectiveness.

Studies involving mixed populations were retained if adult ICU survivors were identifiable, even if outcomes for the ICU subgroup were not reported separately. Intervention characteristics and reported outcomes were charted as reported, and findings from the full mixed population were identified as such. These studies contributed to mapping technology use, outcome measures, and implementation or user experience evidence. Findings available only for the mixed population were not interpreted as ICU-specific estimates of clinical effectiveness.

Reports arising from the same parent study were linked during synthesis and retained when they contributed distinct evidence. Each parent study was counted once when determining the number of unique studies. No formal methodological quality or risk-of-bias appraisal was conducted because the purpose of this scoping review was to map the breadth, characteristics, and gaps of the available evidence rather than to assess the certainty of intervention effects.

## Results

### Study Selection

The updated database searches identified 2557 records. After the removal of 1291 duplicates, 1266 records underwent title and abstract screening, of which 906 were excluded. Full texts were sought for 360 reports; 5 could not be retrieved, leaving 355 reports for eligibility assessment. Of these, 328 were excluded because of an ineligible study design (n=81), an ineligible population (n=85), the absence of an eligible digital health intervention (n=64), or the absence of a mobility-related outcome (n=98). Ultimately, 27 reports representing 24 unique parent studies were included in the review. The study selection process is presented in the PRISMA flow diagram (Figure 1).

**Figure 1.**
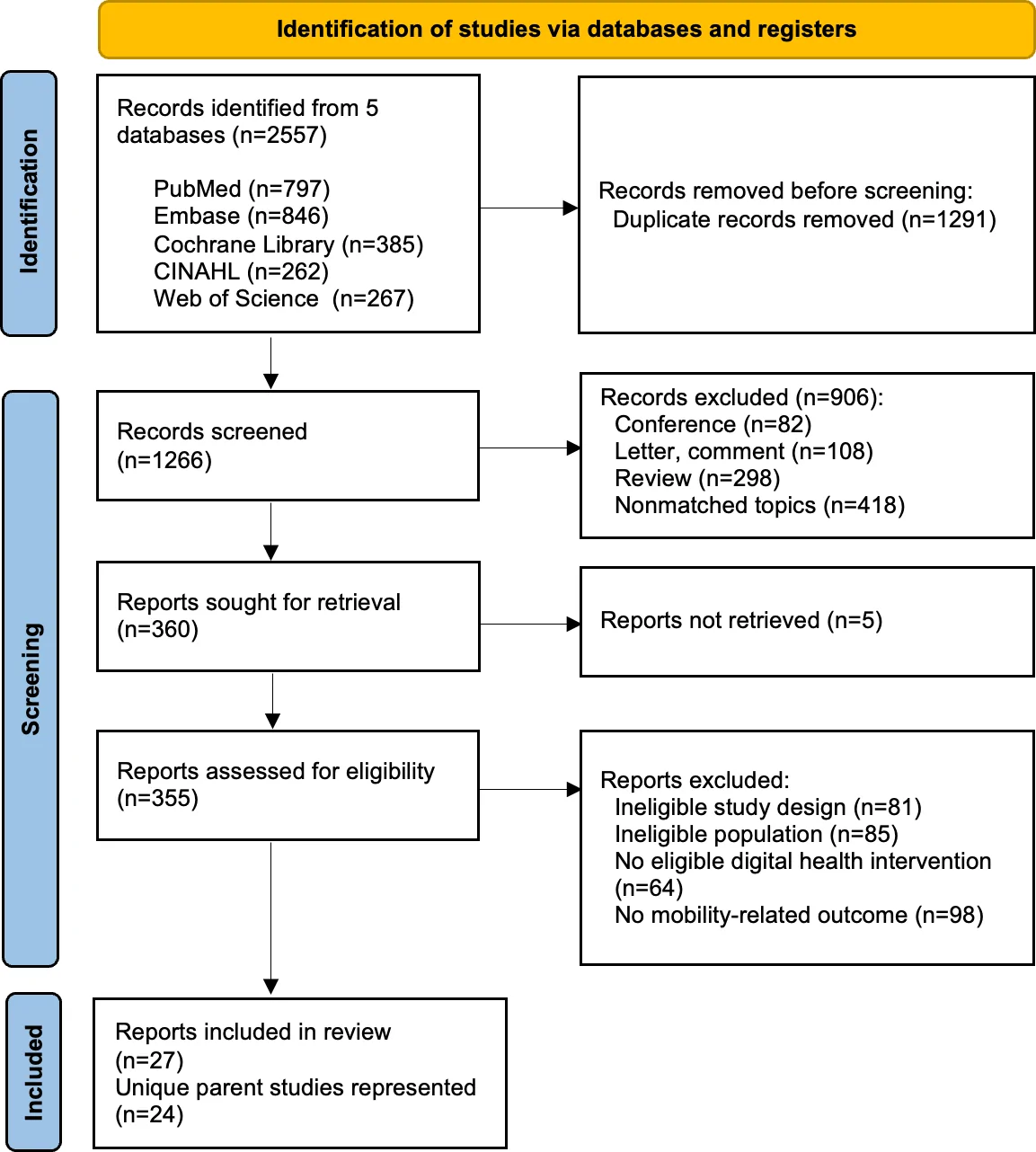
PRISMA (Preferred Reporting Items for Systematic Reviews and Meta-Analyses) flowchart presenting the search outcomes and study selection process.

### Study Characteristics

The review included 27 reports representing 24 unique parent studies, comprising 11 randomized trial reports [39-49], 9 nonrandomized quantitative reports [50-58], 3 qualitative reports [59-61], 2 case reports [62,63], 1 innovation report [64], and 1 multiple methods report [65]. Reports arising from the same parent study were linked and retained when they contributed distinct evidence; each parent study was counted once in the total number of unique studies. The 3 pairs of linked reports were O’Neill et al [44] and Ghiglieri et al [61], Kovaleva et al [59] and Boehm et al [49], and Rose et al [64] and Apps et al [65].

The studies were conducted across diverse geographical settings and included survivors of COVID-19–related critical illness [39-41,43,45,47,51-53,55,62], acute respiratory failure [42,48,54], sepsis and/or acute respiratory distress syndrome [46,49,59], prolonged mechanical ventilation [44,50,61,65], and broader post-ICU populations [48,56-58,60], as well as a survivor of severe burn injury requiring prolonged critical care [63]. Participant characteristics and age were reported heterogeneously across studies. Basic study characteristics are summarized in Table 2, while detailed information on the study populations, digital health technologies, intervention components, duration, delivery format, providers, settings, and mobility-related outcomes is provided in Table S1 in Multimedia Appendix 1.

**Table 2.** Characteristics of included study reports.^a^

Study	Country	Study design	Sample size	Age, y
de Vries et al [50], 2025	Netherlands	Feasibility study	n=10	Median 71 (IQR 63-79) [64-80]
Balakrishnan et al [39], 2023	United States	Exploratory randomized controlled trial	I^b^=20; C^c^=20	Mean (SD) for I: 47.0 (13.9); C: 50.2 (11.5)
Capin et al [40], 2022	United States	Pilot randomized feasibility study	I=29; C=15	Mean (SD) for I: 52.0 (10.0); C: 53.0 (10.0)
Şahin et al [41], 2023	Türkiye	Randomized controlled trial	I=21; C=21	Mean (SD) for I: 57.7 (8.4); C: 63.7 (7.9)
Khan et al [42], 2024	United States	Randomized controlled trial	I=233; C=233	Mean (SD) for I: 55.2 (14.3); C: 56.9 (14.4)
Trzmiel et al [43], 2023	Netherlands	Randomized controlled trial	I=42; C=39	Mean (SD) for I: 67.4 (8.5); C: 64.9 (11.7)
O’Neill et al [44], 2026	United Kingdom	Pragmatic multicenter randomized clinical trial	I=231; C=198	Mean (SD) for I: 55.0 (14.0); C: 55.0 (13.8)
Mayer et al [51], 2021	United States	Pragmatic pre-post comparative study	I=22; C=10	Median for I: 56 [53-62]; C: 55 [45-67]
Carpallo-Porcar et al [45], 2025	Spain	Pilot randomized controlled trial	I=17; C=18	Mean (SD) for I: 58.0 (2.0); C: 59.0 (9.0)
Howroyd et al [52], 2023	United Kingdom	Service evaluation study	n=28	Mean (SD) for 54.0 (11.0)
Hunter et al [53], 2022	United Kingdom	Multicenter observational study	n=50	Median for MDT^d^ site: mean 61 (range 49-73); Control site: mean 57 (range 35-77)
Ramalingam et al [62], 2020	Singapore	Case report	n=1	64
Rose et al [64], 2022	United Kingdom	Innovation report	n=19 enrolled	Median 57
Taylor et al [46], 2022	United States	Multicenter pragmatic randomized clinical trial	I=349; C=342	Mean (SD) for I: 64.7 (14.9); C: 62.7 (15.5)
Kovaleva et al [59], 2023	United States	Qualitative descriptive study	Patients (n=14), caregivers (n=12)	Not reported
Parker et al [54], 2020	United States	Prospective usability and acceptability study	Patient-caregiver dyads (n=10)	Median 50.5 [41-65]
Vlake et al [47], 2022	Netherlands	Multicenter randomized controlled trial	I=44; C=45	Median for I: 61 [55-66]; C: 59 [52-66]
Martin et al [55], 2021	Belgium	Prospective observational comparative study	I=14; C=13	Mean (SD) for I: 60.8 (10.4); C: 61.9 (10.7)
Zacharelou et al [60], 2025	International (Europe)	International qualitative focus group study	n=22	Range 35‐75
Kim et al [56], 2021	Canada	Prospective observational study	n=12	Mean (SD): 66.0 (8.12)
Cha et al [57], 2026	South Korea	Prospective single-arm pilot study	n=25 enrolled; n=20 analyzed	Mean (SD): 52.7 (13.9)
Fortune et al [58], 2026	United States	Prospective observational substudy nested within a randomized clinical trial	n=52 enrolled; n=51 with valid activity-monitor data	Median for I: 67 [65-71]; C: 64 [54-71]
Rosa et al [48], 2026	Brazil	Stepped-wedge cluster randomized controlled trial	n=1916 randomized (I=1063; C=853); n=1687 in primary analysis	Mean 60.6 (SD 17.3)
Lathrop et al [63], 2026	Chile	Case report	n=1	24
Boehm et al [49], 2026	United States	Pilot feasibility randomized controlled trial	n=91 randomized (I=46; C=45)	Median for I: 57 [39-67]; C: 56 [47-68]
Ghiglieri et al [61], 2026	United Kingdom	Theory-informed qualitative interview substudy	ICU survivors, n=35 (RR^e^=20; SC^f^=15); staff, n=6	Mean (SD) for RR: 56.2 (17.0); SC: 66.1 (9.3)
Apps et al [65], 2026	United Kingdom	Multiple methods study	Questionnaire, n=59; interviews, n=30; n=75 unique participants	Median for questionnaire: 58 [48-66]; interviews: 55.5 [49-61]

a Age is reported as mean (SD), median (IQR), or range, as provided in the original studies. Linked reports from the same parent study were retained when they contributed distinct evidence; each parent study was counted once in the number of unique studies.

b I: intervention group.

c C: control or comparator group.

d MDT: multidisciplinary team.

e RR: remote rehabilitation.

f SC: standard care.

### Statistical and Analytical Approaches

Statistical and analytical approaches varied across study designs, ranging from descriptive and conventional group comparisons to adjusted regression, mixed-effects, and longitudinal or clustered models; qualitative studies used content, thematic, or framework-based analyses [40,42,44,48,59,61]. Reported handling of home-generated digital data included synchronization checks, wear-time or data-completeness criteria, exclusion of insufficient observations, and aggregation across valid monitoring periods [39,40,53,56-58], whereas explicit signal-level filtering or cross-device calibration was rarely reported. Detailed study-level information is provided in Table S2 in Multimedia Appendix 1.

### Characteristics of Digital Health Technologies

#### Types of Digital Health Technologies

The included reports covered 7 categories of digital health technologies. Telerehabilitation was the most frequently represented category and included remotely delivered exercise, multicomponent rehabilitation, and rehabilitation support [41,44,45,48,51,52,55,61,62]. Telemedicine approaches primarily involved remote consultation, symptom review, care coordination, and virtual ICU recovery services [39,42,46,49,59]. Other technologies included mobile health (mHealth) apps [40,54,57], wearable monitoring [53,56,58], VR [47,50,63], digital recovery platforms [48,60,65], and robot-assisted rehabilitation [43].

#### Duration and Setting

Intervention duration and timing varied substantially. The shortest approach was a single telemedicine consultation conducted within 14 days after hospital discharge [39], whereas longer-term programs extended to 12 months and incorporated ongoing symptom monitoring, care coordination, or physical activity monitoring [42,53]. Structured rehabilitation programs commonly lasted 6 to 8 weeks [41,44,51,52,61] or approximately 12 weeks [40,45,65]. Other studies used shorter inpatient programs or assessed recovery at predefined postdischarge time points [49,50,57,58].

Home-based delivery was the most common setting and was used for telemedicine, telerehabilitation, mHealth, wearable monitoring, and digital recovery platforms [39-42,44-46,52,53,56,58]. Other technologies were delivered in outpatient or post-ICU follow-up settings [47,51], during inpatient recovery [48,50,57,63], or in specialized rehabilitation facilities [43].

#### Delivery Format

Delivery formats ranged from clinician-led synchronous interactions to asynchronous self-directed technologies. Synchronous videoconferencing or telephone-based delivery was frequently used for telemedicine and telerehabilitation [39,41,44,46,49,52,55,59,62]. Asynchronous or self-directed approaches included mobile apps, web-based recovery platforms, and wearable devices used for exercise guidance, recovery monitoring, goal tracking, or self-management support [40,45,48,53,54,56,58,60,65]. Several interventions adopted hybrid formats combining digital and in-person or multiple remote components [40,42,51,62]. Technology-assisted formats also included therapist- or researcher-assisted immersive VR [47,50,63] and supervised human-robot interaction [43].

#### Health Care Providers Involved

Digital health interventions involved a broad range of health care professionals. Physiotherapists were frequently involved in exercise-based rehabilitation, telecoaching, and technology-assisted rehabilitation [40,41,43,45,51-53,55,57,62,63]. Physicians and ICU clinicians contributed to teleconsultation and virtual ICU recovery services [39,49,59], while nurses were involved in care coordination, transitional care, and recovery support [42,46,47,52]. Several interventions were delivered by multidisciplinary teams involving combinations of physicians, physiotherapists, occupational therapists, psychologists, pharmacists, rehabilitation professionals, and other recovery specialists [48,49,53,59,62]. Other approaches relied partly on self-directed use of mobile apps or wearable devices with limited direct professional supervision [54,56,58].

#### Functional Components of Digital Health Technologies

Exercise-based rehabilitation was the most frequently represented intervention component. These programs incorporated aerobic or endurance exercise, resistance or strengthening exercises, balance training, respiratory exercises, upper-limb training, functional activity, and individualized mobility exercises delivered through digital or technology-assisted formats [40,41,43-45,50-52,55,57]. Monitoring and assessment represented another major function and included remote review of vital signs or symptoms, wearable-based monitoring of physical activity, and digital tracking of recovery progress [39,40,42,46,54,56,58,64,65]. Educational resources, goal setting, and self-management support were incorporated in several interventions [45,52,54,60,62,64,65], while teleconsultation, care coordination, psychological support, and peer support extended the role of digital technologies beyond exercise delivery alone [39,42,44,46,49,59,61].

Explicit use of named theoretical models or frameworks was uncommon. Khan et al [42] described the mobile critical care recovery program as a collaborative care model, while Apps et al [65] reported a theoretically informed recovery goal menu structured according to the Canadian model of occupational performance and engagement and used the theoretical framework of acceptability (TFA) as an analytic lens. Ghiglieri et al [61] also explicitly used the TFA to guide the qualitative study and analysis. Other studies incorporated theory-informed strategies or behavioral constructs, including biobehaviorally informed lifestyle coaching and motivational interviewing [40], self-efficacy and motivation for physical activity [45], and individualized goal setting with Goal Attainment Scaling [64], but did not explicitly report a named overarching theoretical framework guiding intervention design. Overall, explicit theoretical grounding was inconsistently reported across the included evidence.

Table 3 summarizes the 7 digital health technology categories and their principal characteristics. Figure 2 presents the distribution of these technologies across the ICF domains of body functions and structures, activities, and participation.

**Table 3.** Summary of digital health technology categories and ICF-mapped mobility outcomes after critical illness.^a^

Digital health technology	Principal functions and delivery	ICF^b^ mobility domains and representative outcomes
Telerehabilitation (n=9)	Remote exercise training, telecoaching, multicomponent rehabilitation, education, goal setting, and peer support; delivery: video, telephone, mobile apps, or hybrid formats	Body functions and structures: muscle strength and fatigue; activities: 6MWT^c^, STST^d^, SPPB^e^, TUG^f^, disability, and physical-dependence measures; participation: return to work
Telemedicine (n=5)	Remote consultations, symptom review, medication management, care coordination, and virtual ICU recovery clinics; delivery: video, telephone, and electronic health support	Activities: 6MWT, ADL^g^, IADL^h^, and functional status
Mobile health (n=3)	App-guided exercise, rehabilitation education, goal tracking, symptom or mood monitoring, and wearable integration; delivery: mobile apps with self-directed or hybrid support	Body functions and structures: MRC-SS^i^ and handgrip strength; activities: STST, TUG, ICU Mobility Scale, and FAC^j^
Wearable monitoring (n=3)	Continuous or intermittent monitoring of physical activity and free-living performance; delivery: wrist-worn or consumer-grade wearable devices	Activities: daily step count, active time, cadence, 6MWT, and ADL
Virtual reality (VR; n=3)	Immersive rehabilitation, rehabilitation gaming, and ICU^k^ recovery education; delivery: therapist-assisted or in-person immersive VR	Body functions and structures: handgrip strength; activities: MMI^l^, FIM^m^, and ambulation
Robot-assisted rehabilitation (n=1)	Robot-assisted therapeutic exercise; delivery: supervised human-robot interaction	Body functions and structures: handgrip strength; activities: 6MWT, balance performance, and FIM
Digital recovery platforms (n=3)	Goal setting, recovery monitoring, education, digital diaries, reminders, communication, and peer support; delivery: interactive web- or app-based platforms	Activities: ADL and functional independence; participation: activity participation and recovery goals explicitly related to meaningful life activities

a Each report was assigned to one primary technology category according to its predominant digital function and mode of delivery; counts represent reports rather than unique parent studies. A report could contribute to multiple ICF domains when distinct mobility-related outcomes supported each assignment.

b ICF: International Classification of Functioning, Disability and Health.

c 6MWT: 6-minute walk test.

d STST: sit-to-stand test.

e SPPB: short physical performance battery.

f TUG: Timed Up and Go test.

g ADL: activity of daily living.

h IADL: instrumental activity of daily living.

i MRC-SS: Medical Research Council Sum Score.

j FAC: functional ambulation category.

k ICU: intensive care unit.

l MMI: Morton Mobility Index.

m FIM: functional independence measure.

**Figure 2.**
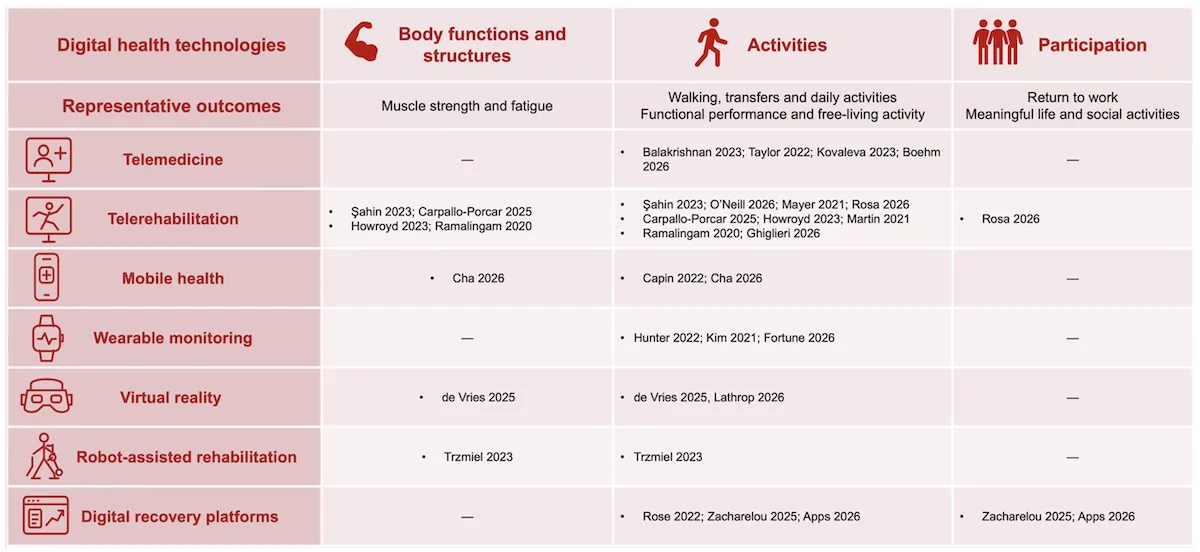
Digital health technologies and ICF domains among ICU survivors. Each report was assigned to one primary technology category according to its predominant digital function and mode of delivery; for multicomponent interventions, the principal component delivering rehabilitation or recovery support determined the category. Each distinct mobility-related outcome was mapped to the ICF domain it most directly represented: body functions and structures, activities, or participation. A report could appear in more than one domain when it reported distinct outcomes representing those domains. Cell entries indicate outcome coverage, including qualitative findings, rather than intervention effectiveness; reports without a directly mappable mobility outcome are not shown in the matrix. Self-management, feasibility, and acceptability were not coded as mobility domains [39-62]. ICF: International Classification of Functioning, Disability and Health; ICU: intensive care unit.

### Mobility Assessment and Mobility-Related Findings

#### Mobility Assessment Tools

A wide range of mobility-related measures was reported across the included studies, reflecting substantial heterogeneity in the assessment of post-ICU recovery. The 6MWT was the most frequently reported assessment tool (n=7) [39,41,43,45,51,58,62], followed by sit-to-stand tests, including the 30-second and 1-minute sit-to-stand tests, which were used to assess functional exercise capacity (n=5) [40,44,45,52,55].

Within the ICF domain of body functions and structures, muscle strength was assessed primarily using handgrip strength [41,43,50,57,62], with additional measures including the Medical Research Council Sum Score and Arm Curl Test. Activity-level outcomes were more diverse and included the 6MWT, sit-to-stand tests, Timed Up and Go test, Short Physical Performance Battery, de Morton Mobility Index, functional independence measure, Berg Balance Scale, ICU Mobility Scale, functional ambulation category, daily step count, ADLs, instrumental ADLs, disability, and physical dependence. Wearable-based measures of free-living activity, including daily step count, active time, and cadence, were reported by Hunter et al [53], Kim et al [56], and Fortune et al [58].

Participation-level outcomes were infrequently assessed. Rosa et al [48] assessed return to work; Zacharelou et al [60] qualitatively explored participation in meaningful daily, community, or social activities; and Apps et al [65] reported recovery goals explicitly related to meaningful life roles or participation. Overall, mobility assessment focused predominantly on body functions and activities, with limited coverage of participation.

#### Mobility-Related Clinical Findings

Evidence from controlled and comparative studies was mixed (Table S3 in Multimedia Appendix 1). Among studies with clinical primary outcomes, the 12-month mobile critical care recovery program did not significantly improve the SF-36 physical component summary compared with control [42]. Remote multicomponent rehabilitation did not significantly improve the primary EQ-5D-5L utility outcome at 8 weeks [44]. ICU-specific VR did not demonstrate consistent benefits in its primary psychological or health-related quality-of-life outcomes [47]. In the stepped-wedge trial by Rosa et al [48], overall EQ-5D-3L utility at 90 days was higher in the intervention group, but this difference was not significant when the analysis was restricted to survivors.

Findings for secondary mobility-related outcomes were also inconsistent. For the secondary 30-second sit-to-stand outcome, O’Neill et al [44] reported a significant adjusted between-group difference at 8 weeks favoring remote rehabilitation. In the robot-assisted rehabilitation trial, a significant group × time interaction was observed for the functional independence measure motor subscale, whereas consistent between-group advantages were not demonstrated across other functional measures [43]. Rosa et al [48] found no significant between-group differences in new disability, physical dependence, or return to work. Similarly, no clear between-group mobility benefit was demonstrated in the telemedicine study by Balakrishnan et al [39] or in the telehealth vs in-person rehabilitation comparison by Mayer et al [51]. In the observational study by Hunter et al [53], step counts increased more at the multidisciplinary-team site than at control sites, although the nonrandomized design limits causal interpretation. Noncomparative studies reported improvements over time in mobility, exercise capacity, physical activity, or functional independence [50,52,57,58,62,63]. These findings were used to describe recovery trajectories and outcome coverage rather than as evidence of intervention effectiveness.

### Implementation Feasibility and User Acceptability

Evidence on feasibility, safety, adherence, usability, acceptability, and user experience was synthesized separately from clinical outcome evidence. Across the included reports, these outcomes were assessed using measures of recruitment and retention, intervention completion and adherence, safety, usability and satisfaction, and qualitative accounts of user experience and implementation barriers.

### Feasibility, Safety, and Adherence

Feasibility, safety, adherence, and implementation outcomes were synthesized separately from clinical effectiveness findings (Table S4 in Multimedia Appendix 1). Several interventions reported favorable engagement or completion, although substantial variation was observed across studies. Remote multicomponent rehabilitation achieved full adherence in 73.2% (169/231) of participants, with 88.7% (205/231) attending at least one session [44], while virtual post-ICU rehabilitation showed 73.6% (28/38) retention and 82% (23/28) adherence [52]. Wearable-monitoring studies demonstrated the feasibility of collecting home activity data, but incomplete device use, technical failures, and missing data reduced data completeness [53,56,58]. In the multicenter stepped-wedge trial, among 300 eligible intervention patients, 269 (89.7%) attended at least 1 ward session and 222 (74.0%) attended at least 1 postdischarge session [48]. Thus, digital interventions were generally deliverable, but sustained participation and completeness of remotely generated data remained variable.

### Acceptability, Usability, and User Experience

Acceptability and user experience findings were generally favorable but were interpreted separately from clinical effectiveness (Table S5 in Multimedia Appendix 1). Remote multicomponent rehabilitation received higher acceptability ratings than standard care [44], while a mobile app prototype achieved median System Usability Scale scores of 76 among ICU survivors and 88 among caregivers [54]. ICU-specific VR was associated with higher satisfaction with ICU aftercare [47], and favorable satisfaction or acceptability was also reported for app-based rehabilitation, immersive VR, telemedicine recovery clinics, and digital recovery platforms [49,57,63,65]. Qualitative findings highlighted individualized support, continuity of care, reassurance, and professional access as facilitators, whereas technical burden, limited digital access, and insufficient personalization were recurrent barriers [59,61,65].

## Discussion

### Principal Findings

This scoping review mapped the use of digital health technologies to support mobility recovery among ICU survivors across 7 technology categories. Interventions most commonly incorporated exercise-based rehabilitation, alongside monitoring, education, self-management support, and remote follow-up. Mobility outcomes were heterogeneous and concentrated on body functions and activity-level performance, with comparatively limited attention to participation. Although feasibility and acceptability were generally favorable when assessed, evidence of clinical effectiveness remained mixed, and long-term implementation data were limited.

### Remote and Telehealth-Based Technologies as a Major Strategy for Post-ICU Recovery

A notable finding of this review was that telemedicine and telerehabilitation were the most frequently adopted digital health technologies. This predominance may reflect the substantial and persistent rehabilitation needs of ICU survivors, who commonly experience long-term physical impairment, reduced exercise capacity, respiratory dysfunction, and difficulties accessing postdischarge rehabilitation services [66-68]. Digital technologies offer an opportunity to extend rehabilitation beyond hospital settings and provide continuous support throughout the recovery trajectory [69]. In addition to improving access to rehabilitation, remote and telehealth-based approaches may reduce geographical and transportation barriers, facilitate continuity of care after hospital discharge, enable timely monitoring of recovery, and strengthen patient self-management in home settings, where access to structured post-ICU rehabilitation is often limited.

The COVID-19 pandemic further accelerated the adoption of remote rehabilitation technologies. Eleven of the 24 (45.8%) included studies focused on survivors of COVID-19–related critical illness, reflecting the urgent demand for scalable rehabilitation strategies during a period when infection-control measures substantially restricted conventional outpatient and community-based rehabilitation services. At the same time, the rapid expansion of telehealth infrastructure and increasing familiarity with digital health care delivery further facilitated the implementation of remote rehabilitation programs. Together, these factors contributed to the widespread use of telemedicine and telerehabilitation for supporting patients with persistent physical and respiratory impairments following severe COVID-19 [70].

However, the predominance of COVID-19–related studies should be considered when interpreting the current evidence. Compared with general ICU survivors, individuals recovering from COVID-19 may experience distinct recovery patterns, particularly persistent respiratory symptoms, postviral fatigue, and impaired exercise tolerance, which may influence both rehabilitation needs and responses to digital intervention [71,72]. In contrast, evidence involving non-COVID ICU populations, such as survivors of sepsis, acute respiratory failure, prolonged mechanical ventilation, and other critical illnesses, remains relatively limited. Furthermore, many COVID-19–related digital programs were rapidly developed during the pandemic and were commonly evaluated over short intervention periods, with limited evidence regarding their long-term sustainability and integration into routine post-ICU care [70,73]. Beyond these population-specific considerations, successful implementation of remote rehabilitation also depends on patients’ digital literacy, access to internet-enabled devices, reliable connectivity, and, in some cases, caregiver support. These factors may influence patient engagement, equity of access, and the scalability of digital rehabilitation programs in routine clinical practice.

### Exercise-Based Rehabilitation Remains the Core Functional Component of Digital Health Technologies

Exercise-based rehabilitation emerged as the most frequently reported functional component among the included digital health technologies. This finding is unsurprising given that persistent physical impairments, including muscle weakness, reduced exercise tolerance, and limitations in physical functioning, are among the most common consequences experienced by ICU survivors after critical illness. Previous studies have shown that these impairments may persist for months or even years after hospital discharge and contribute to long-term disability, reduced health-related quality of life, and difficulties returning to previous life roles [22-24,74]. Consequently, exercise rehabilitation remains a cornerstone of post-ICU recovery programs and is strongly recommended to mitigate long-term physical disability and promote functional restoration [75]. This predominance may also reflect the characteristics of current digital health technologies, as exercise programs are more readily delivered through telehealth platforms, and their outcomes can be objectively monitored using wearable devices and standardized performance measures. By contrast, broader domains such as social participation and community reintegration are less amenable to remote measurement and digital monitoring.

However, the predominance of exercise-centered technologies highlights an important gap in current digital rehabilitation strategies. Mobility recovery after critical illness is increasingly recognized as a multidimensional process encompassing body functions and structures, activities, and participation [14,76-78]. Consistent with the findings of this review, existing digital health technologies primarily addressed body functions and activity-level performance, particularly exercise capacity, muscle strength, and functional performance, while participation outcomes were less frequently assessed. Therefore, future digital health technologies should move beyond physical rehabilitation alone and incorporate more comprehensive, person-centered recovery goals.

Furthermore, although digital technologies offer opportunities for continuous monitoring and personalized rehabilitation, current apps largely remain tools for remote delivery and assessment rather than adaptive rehabilitation systems. Future integration of wearable sensing, AI, and real-time feedback may facilitate closed-loop rehabilitation models that dynamically tailor interventions according to patients’ changing recovery needs [79,80].

### Emerging Digital Technologies: From Feasibility to Patient-Centered Implementation

Beyond conventional telemedicine and telerehabilitation, emerging digital technologies included VR, rehabilitation robots, wearable activity monitoring, and digital recovery platforms. Current evidence for these technologies was predominantly derived from feasibility, pilot, and observational studies, with generally favorable feasibility and user experience findings when assessed. However, evidence of clinical effectiveness remained limited and inconsistent. VR-based rehabilitation was perceived as enjoyable and feasible among ICU survivors, with no serious adverse events reported [50]. App-based and video-supported rehabilitation programs also reported favorable adherence and retention, supporting their implementation feasibility rather than establishing clinical effectiveness [40,52]. In addition, digital recovery platforms and virtual follow-up services were valued for facilitating access to information, self-management, and communication with health care professionals [54,59,60,64].

However, the current evidence primarily reflects early-stage implementation, as many emerging digital technologies were evaluated in feasibility studies, pilot trials, or small observational studies with relatively short follow-up periods. Although several studies reported favorable usability and acceptability through satisfaction questionnaires, usability scales, or qualitative interviews, a comprehensive understanding of long-term user engagement and implementation barriers remains limited. Future development of digital rehabilitation technologies should therefore adopt more patient-centered approaches, including co-design involving ICU survivors and caregivers, as well as mixed methods evaluations that integrate quantitative usability measures with qualitative exploration of user experiences. In addition, successful implementation will require attention to data privacy and security, integration into routine clinical care, and adequate training and support for health care professionals. Such considerations may help ensure that emerging digital technologies are not only technically feasible but also meaningful, accessible, and sustainable in supporting long-term recovery after critical illness.

### Heterogeneity and Narrowness of Mobility Outcome Measurement

Another important finding of this review was the substantial heterogeneity in mobility assessment and outcome measurement. Most studies focused on basic physical capacity and functional performance, commonly assessed using the 6MWT, sit-to-stand tests, handgrip strength, the Timed Up and Go test, and other performance-based measures [39-41,43-45,51,52,55,62]. Although these outcomes are clinically meaningful for evaluating physical recovery after critical illness, they capture only part of the multidimensional nature of mobility.

Measures addressing broader activity and participation outcomes were less frequently reported, including functional independence [43,62], ADLs, activity participation, and mobility- or participation-related recovery goals [42,43,54,60,62,64]. This imbalance suggests that current digital health research has predominantly evaluated mobility through body functions and activity-level performance rather than across the full spectrum of post-ICU functioning.

The heterogeneity of outcome measurement remains a major challenge for comparing and synthesizing evidence across digital rehabilitation studies. The PRACTICE (Physical Rehabilitation Core Outcomes in Critical Illness) core outcome set provides a framework to promote standardized reporting of physical rehabilitation outcomes in critically ill populations [81]. However, as digital health interventions increasingly extend beyond exercise training to include remote monitoring, self-management, and long-term recovery support, future research should combine standardized core outcomes with broader multidimensional assessments that reflect independence, participation, and patient-centered recovery trajectories. Analytical approaches were also heterogeneous, ranging from descriptive and unadjusted comparisons to adjusted regression, mixed-effects, and longitudinal or clustered models. This variation reflected differences in study design but also limited the comparability of effect estimates across studies. Future studies should prespecify analytical strategies appropriate to repeated and longitudinal measurements and consistently report effect estimates with measures of uncertainty.

The handling of home-generated digital data was also inconsistently reported. Reported approaches included synchronization checks, wear-time or data-completeness thresholds, exclusion of insufficient observations, aggregation across valid monitoring periods, and missing-data assessment, whereas explicit signal-level artifact filtering and cross-device calibration were rarely described. Future studies should prespecify device validation, valid-wear and nonwear criteria, data-cleaning procedures, and missing-data handling to improve the reproducibility and interpretability of remotely generated data.

### Implications for Future Research

Several implications for future research emerged from this review. First, more adequately powered randomized controlled trials with longer follow-up periods are needed to establish the long-term effectiveness and sustainability of digital health technologies, given that mobility recovery after critical illness is a prolonged and dynamic process. Second, future studies should move beyond short-term physical outcomes and incorporate multidimensional recovery indicators, including independence in ADLs, community and social participation, return to occupational or caregiving roles, patient-reported physical function, and individualized mobility- or participation-related recovery goals. Third, theoretical foundations were infrequently and inconsistently reported; future interventions would benefit from clearly articulated behavioral or rehabilitation frameworks where appropriate. Finally, future research should consider implementation factors, including digital literacy, technology accessibility, caregiver involvement, and health equity, to support broader adoption in ICU survivorship care.

### Strengths and Limitations

This review has several strengths. A comprehensive search strategy was applied, and evidence was synthesized across technology types, delivery models, intervention components, and mobility-related outcomes. The review also identified important gaps in outcome measurement, theoretical grounding, and implementation evidence.

Several limitations should be acknowledged. First, although 27 reports representing 24 unique parent studies were included, many were feasibility, pilot, qualitative, or small observational studies, limiting conclusions regarding clinical effectiveness. Second, substantial heterogeneity in populations, interventions, outcome measures, and analytical approaches limited direct comparisons across studies. Third, a substantial proportion of the evidence was derived from survivors of COVID-19–related critical illness, which may limit generalizability to the broader ICU survivor population. In addition, classifying multicomponent interventions according to their predominant digital function may have simplified the overlap between technology categories. Finally, no formal methodological quality or risk-of-bias appraisal was conducted; therefore, differences in study quality were not incorporated into the synthesis, and findings regarding clinical effectiveness should be interpreted cautiously.

### Conclusions

Digital health technologies have been applied across diverse approaches to support mobility recovery among ICU survivors, with telemedicine and telerehabilitation being the most frequently used. Current evidence predominantly addresses body functions and activity-level performance, with limited attention to participation. Although feasibility and acceptability were generally favorable, evidence of clinical effectiveness remains inconsistent, highlighting the need for rigorous longitudinal studies using multidimensional, patient-centered mobility outcomes.

## Supplementary material

10.2196/105445 Multimedia Appendix 1 Search strategy and supplementary tables (Tables S1-S5) for the scoping review.

10.2196/105445 Checklist 1 PRISMA-ScR checklist.

## Abbreviations

6MWT: 6-minute walk test
ADL: activity of daily living
ICF: International Classification of Functioning, Disability, and Health
ICU: intensive care unit
mHealth: mobile health
PCC: population, concept, and context
PRACTICE: Physical Rehabilitation Core Outcomes in Critical Illness
PRISMA-ScR: Preferred Reporting Items for Systematic Reviews and Meta-Analyses Extension for Scoping Reviews
SF-36: 36-item Short Form Health Survey
TFA: theoretical framework of acceptability
VR: virtual reality
