# Supplementary material for: Digital Health Technologies for Mobility Recovery Among Intensive Care Unit Survivors: Scoping Review
Source: J Med Internet Res. 2026 Nov 5;28:e105445. doi: 10.2196/105445 (PMC13739207; doi:10.2196/105445)
Supplement: Supplementary file 1 [file jmir-v28-e105445-s001.docx]

[Search strategy	1](#_Toc241415980)

[Table S1. Characteristics of Digital Health Technologies and Interventions	7](#_Toc241415981)

[Table S2. Statistical and Analytical Approaches and Handling of Home-Generated Digital and Device Data	15](#_Toc241415982)

[Table S3. Primary and Secondary Clinical Outcome Findings in Controlled and Comparative Studies	18](#_Toc241415983)

[Table S4. Feasibility, Adherence, Safety, and Implementation Findings of Digital Health Interventions	22](#_Toc241415984)

[Table S5. Acceptability outcomes and findings of digital health interventions	25](#_Toc241415985)

# Search strategy

Database: **PubMed**

Final search date: September 21, 2026

Publication date limit: January 1, 2016, to September 21, 2026, applied using the PubMed publication date filter.

Language filter used in database: None

Study-design/publication-type filter used in database: None

Records retrieved in updated search:**797**

| **No.** | **Query** | **Result** |
| --- | --- | --- |
| #1 | Intensive Care Units [MeSH Terms] | 126,463 |
| #2 | Critical illness [MeSH Terms] | 46,535 |
| #3 | Survivors [MeSH Terms] | 52,016 |
| #4 | "intensive care unit*"[Title/Abstract] OR "ICU"[Title/Abstract] OR "critically ill"[Title/Abstract] OR "post-ICU"[Title/Abstract] OR "ICU survivor*"[Title/Abstract] OR "post intensive care syndrome"[Title/Abstract] OR "PICS"[Title/Abstract] | 288,891 |
| #5 | #1 OR #2 OR #3 OR #4 | 382,104 |
| #6 | "Digital Health"[MeSH Terms] OR "Telemedicine"[MeSH Terms] OR "Wearable Electronic Devices"[MeSH Terms] OR "Smartphone"[MeSH Terms] OR "Mobile Applications"[MeSH Terms] OR "Virtual Reality"[MeSH Terms] OR "Artificial Intelligence"[MeSH Terms] OR "Accelerometry"[MeSH Terms] | 430,465 |
| #7 | "digital health"[Title/Abstract] OR "mobile health"[Title/Abstract] OR "mHealth"[Title/Abstract] OR "eHealth"[Title/Abstract] OR "telemedicine"[Title/Abstract] OR "telehealth"[Title/Abstract] OR "telerehabilitation"[Title/Abstract] OR "tele-rehabilitation"[Title/Abstract] OR "remote rehabilitation"[Title/Abstract] OR "remote rehab*"[Title/Abstract] OR "remote monitoring"[Title/Abstract] OR "digital intervention*"[Title/Abstract] OR "digital therapeutics"[Title/Abstract] OR "wearable*"[Title/Abstract] OR "wearable device*"[Title/Abstract] OR "smartwatch*"[Title/Abstract] OR "activity tracker*"[Title/Abstract] OR "fitness tracker*"[Title/Abstract] OR "accelerometer*"[Title/Abstract] OR "smartphone*"[Title/Abstract] OR "mobile application*"[Title/Abstract] OR "mobile app*"[Title/Abstract] OR "virtual reality"[Title/Abstract] OR "VR"[Title/Abstract] OR "artificial intelligence"[Title/Abstract] OR "AI"[Title/Abstract] | 428,957 |
| #8 | #6 OR #7 | 698,150 |
| #9 | "Early Ambulation"[MeSH Terms] OR "Activities of Daily Living"[MeSH Terms] OR "Walking"[MeSH Terms] OR "Dependent Ambulation"[MeSH Terms] OR "Physical Functional Performance"[MeSH Terms] | 216,958 |
| #10 | "mobility"[Title/Abstract] OR "physical function"[Title/Abstract] OR "functional recovery"[Title/Abstract] OR "physical activity"[Title/Abstract] OR "ambulation"[Title/Abstract] OR "activities of daily living"[Title/Abstract] OR "ADL"[Title/Abstract] OR "muscle strength"[Title/Abstract] OR "muscle mass"[Title/Abstract] OR "frailty"[Title/Abstract] OR "post-ICU physical status"[Title/Abstract] OR "early mobilization"[Title/Abstract] OR "early ambulation"[Title/Abstract] OR "accelerated ambulation"[Title/Abstract] | 622,072 |
| #11 | #9 OR #10 | 774,703 |
| #12 | #5 AND #8 AND #11 | 797 |

Database: **Cochrane database**

Final search date: September 21, 2026

Publication date limit: January 1, 2016, to September 21, 2026, applied using the Cochrane database date filter.

Language filter used in database: None

Study-design/publication-type filter used in database: None

Records retrieved in updated search: **385**

| **No.** | **Query** | Results |
| --- | --- | --- |
| #1 | MeSH descriptor: [Intensive Care Units] explode all trees | 6870 |
| #2 | MeSH descriptor: [Critical Illness] explode all trees | 4112 |
| #3 | MeSH descriptor: [Survivors] explode all trees | 3325 |
| #4 | ("intensive care unit" OR "ICU" OR "critically ill" OR "post-ICU" OR "ICU survivor" OR "post intensive care syndrome" OR "PICS"):ti,ab,kw | 46991 |
| #5 | #1 OR #2 OR #3 OR #4 | 51893 |
| #6 | MeSH descriptor: [Digital Health] explode all trees | 336 |
| #7 | MeSH descriptor: [Telemedicine] explode all trees | 6565 |
| #8 | ("digital health" OR "mobile health" OR "mHealth" OR "eHealth" OR "telemedicine" OR "telehealth" OR "telerehabilitation" OR "tele-rehabilitation" OR "remote rehabilitation" OR "remote monitoring" OR "digital intervention*" OR "digital therapeutics" OR "digital therap*" OR "wearable*" OR "wearable device*" OR "smartwatch*" OR "accelerometer*" OR "smartphone*" OR "mobile application*" OR "mobile app*" OR "app-based" OR "virtual reality" OR "VR" OR "exergam*" OR "exer-gam*" OR "artificial intelligence" OR "AI"):ti,ab,kw | 75176 |
| #9 | #6 OR #7 OR #8 | 75556 |
| #10 | MeSH descriptor: [Early Ambulation] explode all trees | 562 |
| #11 | MeSH descriptor: [Activities of Daily Living] explode all trees | 14135 |
| #12 | MeSH descriptor: [Walking] explode all trees | 8986 |
| #13 | MeSH descriptor: [Dependent Ambulation] explode all trees | 22 |
| #14 | ("mobility" OR "physical function" OR "functional recovery" OR "physical activity" OR "ambulation" OR "activities of daily living" OR "ADL" OR "muscle strength" OR "muscle mass" OR "frailty" OR "post-ICU physical status" OR "early mobilization" OR "early ambulation" OR "accelerated ambulation"):ti,ab,kw | 142794 |
| #15 | #10 OR #11 OR #12 OR #13 OR #14 | 153327 |
| #16 | #5 AND #9 AND #15 | 385 |

Database: **Embase**

Final search date: September 21, 2026

Publication date limit: January 1, 2016, to September 21, 2026, applied using the Embase database date filter.

Language filter used in database: None

Study-design/publication-type filter used in database: None

Records retrieved in updated search: **846**

| No. | Query | Results |
| --- | --- | --- |
| #1 | 'intensive care unit'/exp | 435,395 |
| #2 | 'critical illness'/exp | 44,492 |
| #3 | 'survivor'/exp | 138,794 |
| #4 | 'digital health'/exp OR 'telemedicine'/exp OR 'telerehabilitation'/exp OR 'telehealth'/exp OR 'mobile application'/exp OR 'smartphone'/exp OR 'wearable device'/exp OR 'accelerometer'/exp OR 'virtual reality'/exp OR 'artificial intelligence'/exp | 531,696 |
| #5 | 'telemedicine'/exp OR 'telehealth'/exp OR 'telerehabilitation'/exp | 133,881 |
| #6 | 'early ambulation'/exp | 59,177 |
| #7 | 'activities of daily living'/exp | 154,613 |
| #8 | 'walking'/exp | 177,855 |
| #9 | 'dependent ambulation'/exp | 21,018 |
| #10 | 'intensive care unit':ti,ab,kw OR 'icu':ti,ab,kw OR 'critically ill':ti,ab,kw OR 'post-ICU':ti,ab,kw OR 'ICU survivor*':ti,ab,kw OR 'post intensive care syndrome':ti,ab,kw OR 'PICS':ti,ab,kw | 456,625 |
| #11 | 'digital health':ti,ab,kw OR 'mobile health':ti,ab,kw OR 'mHealth':ti,ab,kw OR 'eHealth':ti,ab,kw OR 'telemedicine':ti,ab,kw OR 'telehealth':ti,ab,kw OR 'telerehabilitation':ti,ab,kw OR 'tele-rehabilitation':ti,ab,kw OR 'remote rehabilitation':ti,ab,kw OR 'remote rehab*':ti,ab,kw OR 'remote monitoring':ti,ab,kw OR 'digital intervention*':ti,ab,kw OR 'digital therapeutics':ti,ab,kw OR 'digital therap*':ti,ab,kw OR 'wearable*':ti,ab,kw OR 'wearable device*':ti,ab,kw OR 'consumer wearable*':ti,ab,kw OR 'smartwatch*':ti,ab,kw OR 'accelerometer*':ti,ab,kw OR 'smartphone*':ti,ab,kw OR 'mobile application*':ti,ab,kw OR 'mobile app*':ti,ab,kw OR 'app-based':ti,ab,kw OR 'virtual reality':ti,ab,kw OR 'VR':ti,ab,kw OR 'exergam*':ti,ab,kw OR 'exer-gam*':ti,ab,kw OR 'artificial intelligence':ti,ab,kw OR 'AI':ti,ab,kw | 560,839 |
| #12 | 'mobility':ti,ab,kw OR 'physical function':ti,ab,kw OR 'functional recovery':ti,ab,kw OR 'physical activity':ti,ab,kw OR 'ambulation':ti,ab,kw OR 'activities of daily living':ti,ab,kw OR 'ADL':ti,ab,kw OR 'muscle strength':ti,ab,kw OR 'muscle mass':ti,ab,kw OR 'frailty':ti,ab,kw OR 'post-ICU physical status':ti,ab,kw OR 'early mobilization':ti,ab,kw OR 'early ambulation':ti,ab,kw OR 'accelerated ambulation':ti,ab,kw OR 'early mobility':ti,ab,kw | 857,994 |
| #13 | #1 OR #2 OR #3 OR #10 | 758,692 |
| #14 | #4 OR #5 OR #11 | 735,833 |
| #15 | #6 OR #7 OR #8 OR #9 OR #12 | 1,108,842 |
| #16 | #13 AND #14 AND #15 | 846 |

Database: **CINAHL database**

Final search date: September 21, 2026

Publication date limit: January 1, 2016, to September 21, 2026, applied using the CINAHL database date filter.

Language filter used in database: None

Study-design/publication-type filter used in database: None

Records retrieved in updated search: **262**

| No. | Query | Results |
| --- | --- | --- |
| #1 | SU("Intensive Care Units") | 82,655 |
| #2 | SU("Critical Illness") | 16,794 |
| #3 | SU("Digital Health") OR SU("Telemedicine") OR SU("Telerehabilitation") OR SU("Wearable Electronic Devices") OR SU("Smartphones") OR SU("Mobile Applications") OR SU("Virtual Reality") OR SU("Artificial Intelligence") OR SU("Accelerometers") | 5,343 |
| #4 | SU("Telemedicine") OR SU("Telehealth") OR SU("Telerehabilitation") | 29,565 |
| #5 | TI("intensive care unit*" OR "ICU" OR "critically ill" OR "post-ICU" OR "post-intensive care" OR "PICS" OR "post intensive care syndrome" OR "ICU survivor*" OR "critical illness survivor*" OR "critical care survivor*" OR "ICU discharge*" OR "ICU recover*") | 53,167 |
| #6 | TI("digital health" OR "mobile health" OR "mHealth" OR "eHealth" OR "telemedicine" OR "telehealth" OR "telerehabilitation" OR "tele-rehabilitation" OR "remote rehabilitation" OR "remote rehab" OR "remote monitoring" OR "digital intervention" OR "digital therapeutics" OR "digital therap" OR "wearable" OR "wearable device" OR "consumer wearable*" OR "smartwatch" OR "activity tracker" OR "fitness tracker" OR "accelerometer" OR "smartphone" OR "mobile application" OR "mobile app" OR "app-based" OR "virtual reality" OR "VR" OR "exergam" OR "exer-gam" OR "artificial intelligence" OR "AI" OR "machine learning" OR "deep learning" OR "predictive analytic" OR "adaptive platform*") OR AB("digital health" OR "mobile health" OR "mHealth" OR "eHealth" OR "telemedicine" OR "telehealth" OR "telerehabilitation" OR "tele-rehabilitation" OR "remote rehabilitation" OR "remote rehab" OR "remote monitoring" OR "digital intervention" OR "digital therapeutics" OR "digital therap" OR "wearable" OR "wearable device" OR "consumer wearable" OR "smartwatch" OR "activity tracker" OR "fitness tracker" OR "sensor" OR "accelerometer" OR "smartphone" OR "mobile application" OR "mobile app" OR "app-based" OR "virtual reality" OR "VR" OR "exergam" OR "exer-gam" OR "artificial intelligence" OR "AI") | 111,832 |
| #7 | TI(mobility OR ambulation OR walking OR "physical activity" OR "physical function" OR "functional status" OR "functional recovery" OR "physical recovery" OR "physical performance" OR "exercise capacity" OR "exercise tolerance" OR "6MWT" OR "6-minute walk test" OR frailty OR "muscle strength" OR "activities of daily living" OR ADL) | 52,972 |
| #8 | AB(mobility OR ambulation OR walking OR "physical activity" OR "physical function" OR "functional status" OR "functional recovery" OR "physical recovery" OR "physical performance" OR "exercise capacity" OR "exercise tolerance" OR "6MWT" OR "6-minute walk test" OR frailty OR "muscle strength" OR "activities of daily living" OR ADL) | 455,314 |
| #9 | #1 OR #2 OR #5 | 114,438 |
| #10 | #3 OR #4 OR #6 | 146,417 |
| #11 | #7 OR #8 | 503,274 |
| #12 | #9 AND #10 AND #11 | 262 |

Database: **Web of science**

Final search date: September 21, 2026

Publication date limit: January 1, 2016, to September 21, 2026, applied using the Web of science database date filter.

Language filter used in database: None

Study-design/publication-type filter used in database: None

Records retrieved in updated search: **267**

| **No.** | **Query** | Results |
| --- | --- | --- |
| **#1** | TS=("intensive care unit*" OR ICU OR "critical care" OR "critical illness" OR "critically ill") | 375,282 |
| **#2** | TS=("ICU survivor*" OR "critical care survivor*" OR "critical illness survivor*" OR "post-ICU" OR "post intensive care" OR "post-intensive care" OR "ICU recovery" OR "critical illness recovery" OR PICS OR "post intensive care syndrome" OR "post-intensive care syndrome" OR survivor* OR recover*) | 2,240,071 |
| **#3** | #1 AND #2 | 35,423 |
| **#4** | TS=("digital health" OR "mobile health" OR mHealth OR eHealth OR telemedicine OR telehealth OR telerehabilitation OR "tele-rehabilitation" OR "remote rehabilitation" OR "remote rehab*" OR "virtual rehabilitation" OR "remote monitoring" OR "digital intervention*" OR "digital therap*" OR "digital platform*" OR "online platform*" OR "web-based" OR "internet-based" OR wearable* OR "wearable device*" OR smartwatch* OR "activity tracker*" OR "fitness tracker*" OR accelerometer* OR smartphone* OR "mobile application*" OR "mobile app*" OR "app-based" OR "virtual reality" OR VR OR exergam* OR "robot-assisted rehabilitation" OR videoconferenc*) | 733,482 |
| **#5** | TS=(mobilit* OR "physical function*" OR "physical performance" OR "functional status" OR "functional recover*" OR "physical recover*" OR "physical activit*" OR rehabilitat* OR exercise* OR "exercise capacity" OR walk* OR gait OR ambulation OR "activities of daily living" OR ADL OR "functional independence" OR "muscle strength" OR "handgrip strength" OR "sit-to-stand" OR "6-minute walk*" OR "six-minute walk*") | 2,463,826 |
| **#6** | #3 AND #4 AND #5 | 267 |

# Table S1. Characteristics of digital health technologies and interventions

| **Study** | **Population** | **Digital Health Technology** | **Intervention Components** | **Duration** | **Delivery Format** | **Provider** | **Setting** | **Mobility-Related Outcomes and ICF Domain(s)** | **Explicitly Reported Theory, Model, or Framework** |
| --- | --- | --- | --- | --- | --- | --- | --- | --- | --- |
| de Vries et al [1], 2025 | ICU survivors requiring MV ≥48 h | VR | Upper-limb VR gaming rehabilitation | 20 min/session, 3×/week, up to 4 weeks | In-person assisted digital intervention | Researcher | ICU/general ward | Body functions and structures: handgrip strength; Activities: MMI | Not explicitly reported |
| Balakrishnan et al [2], 2023 | COVID-19 ARDS ICU survivors | Telemedicine | Teleconsultation, 6MWT, and vital-sign review | Single visit within 14 days after discharge | Synchronous video consultation | Intensivist | Home | Activities: 6MWT | Not explicitly reported |
| Capin et al [3], 2022 | COVID-19 ICU survivors | Mobile health (mHealth) | App-based exercise programme with wearable activity monitoring, including aerobic, strength, balance, and breathing training | 12 weeks | Hybrid digital delivery (video + app + wearable) | Physiotherapist | Home | Activities: 30-s STST, TUG | No named overarching model/framework explicitly reported; intervention included biobehaviourally informed lifestyle coaching and motivational interviewing |
| Şahin et al [4], 2023 | COVID-19 ICU survivors | Telerehabilitation | Home exercise programme with remote telecoaching | 8 weeks | Telephone-based synchronous coaching | Physiotherapist | Home | Body functions and structures: handgrip strength; Activities: 6MWT | Not explicitly reported |
| Khan et al [5], 2024 | Survivors of acute respiratory failure | Telemedicine | Remote symptom monitoring, care planning, and multidisciplinary care coordination | 12 months | Hybrid delivery (telephone + home visits) | Nurse care coordinator | Home | No direct ICF mobility outcome: SF-36 PCS reflected broader physical health–related quality of life and was not assigned to a mobility domain | Collaborative care model/approach |
| Trzmiel et al [6], 2023 | COVID-19 ICU survivors | Robot-assisted rehabilitation | Robot-assisted upper-limb exercise training | During rehabilitation programme (45 min/day) | Human–robot interactive delivery | Physiotherapist | Rehabilitation centre | Body functions and structures: handgrip strength; Activities: 6MWT, Berg Balance Scale, FIM | Not explicitly reported |
| O’Neill et al [7], 2026 | ICU survivors requiring MV ≥48 h | Telerehabilitation | Multicomponent rehabilitation including exercise, symptom management, psychological support, and peer support | 6 weeks | Synchronous video or telephone delivery | Trained intervention specialist | Home | Activities: 30-s STST | Not explicitly reported as an overarching intervention framework in the primary trial report; acceptability was examined using TFA in the linked Ghiglieri et al. report |
| Mayer et al [8], 2021 | COVID-19 ICU survivors | Telerehabilitation | Aerobic, resistance, and respiratory muscle training | 8 weeks | Hybrid delivery (telehealth + in-person sessions) | Physiotherapist | Home/outpatient clinic | Activities: 6MWT, SPPB, TUG | Not explicitly reported |
| Carpallo-Porcar et al [9], 2025 | COVID-19 ICU survivors | Telerehabilitation | App-based therapeutic exercise and health education | 12 weeks | Asynchronous app-based rehabilitation with telephone support | Physiotherapist | Home | Body functions and structures: ACT; Activities: 6MWT, 30-s STST | No named overarching model/framework explicitly reported; self-efficacy and motivation were examined as behavioural constructs |
| Howroyd et al [10], 2023 | COVID-19 ICU survivors | Telerehabilitation | Group exercise sessions, peer support, and self-management education | 8 weeks | Synchronous group videoconferencing | Nurse and physiotherapist | Home | Body functions and structures: fatigue; Activities: 1-min STST, physical function | Not explicitly reported |
| Hunter et al [11], 2022 | COVID-19 ICU survivors | Wearable monitoring | Remote monitoring of physical activity and heart rate with optional MDT-supported rehabilitation review | 12 months | Wearable self-monitoring with remote MDT feedback | Physician, physiotherapist, and occupational therapist | Home/community | Activities: daily step count | Not explicitly reported |
| Ramalingam et al [12], 2020 | COVID-19 ICU survivor | Telerehabilitation | Home-based exercise training, education, and multidisciplinary remote follow-up | Post-discharge rehabilitation period | Hybrid delivery (mobile application + videoconferencing) | Rehabilitation physician, physiatrist, physiotherapist, medical social worker | Rehabilitation unit/home | Body functions and structures: handgrip strength; Activities: 6MWT, FIM | Not explicitly reported |
| Rose et al [13], 2022 | ICU survivors | Digital recovery platform | Goal setting, symptom monitoring, education, and communication with ICU recovery team | Not reported | Asynchronous web-based digital platform | ICU recovery coordinator | Post-ICU follow-up | Activities: ADL and activity-related functional recovery goals | No named overarching model/framework explicitly reported; individualized goal setting and Goal Attainment Scaling were used |
| Taylor et al [14], 2022 | ICU sepsis survivors | Telemedicine | Nurse-led transitional care, symptom monitoring, medication review, and care coordination | 30 days | Telephone-based follow-up with electronic health support | Nurse navigator | Home | Activities: functional status; broader post-sepsis recovery outcomes were not independently assigned to an ICF mobility domain | Not explicitly reported |
| Kovaleva et al [15], 2023 | ICU survivors with sepsis/ARDS and caregivers | Telemedicine | Virtual ICU recovery clinic including symptom review, medication management, functional assessment, and caregiver support | Follow-up at 3 and 12 weeks after discharge | Synchronous video consultation | ICU physician, pharmacist, neuropsychologist, multidisciplinary team | Home | Activities: functional status, ADL | Not explicitly reported |
| Parker et al [16], 2020 | Acute respiratory failure survivors and caregivers | Mobile health (mHealth) | Mobile application providing rehabilitation education, goal tracking, mood monitoring, and self-management support | Single usability assessment | Self-directed app-based delivery | Research team | Home | No direct ICF mobility outcome reported: physical rehabilitation was an intervention component rather than a mobility outcome | Not explicitly reported |
| Vlake et al [17], 2022 | COVID-19 ICU survivors | VR | Immersive ICU-VR experience explaining ICU treatment and recovery | Single 14-minute session | In-person VR session | ICU physician and ICU nurse | Post-ICU clinic | No direct ICF mobility outcome: HRQoL and general patient-reported recovery outcomes were not assigned to a mobility domain | Not explicitly reported |
| Martin et al [18], 2021 | COVID-19 ICU survivors | Telerehabilitation | Home-based supervised exercise programme | 3 months | Synchronous remote rehabilitation | Physiotherapist | Home | Activities: 1-min STST, exercise capacity | Not explicitly reported |
| Zacharelou et al [19], 2025 | ICU survivors | Digital recovery platform | Personalised information, e-consultation, peer support, ICU diary, reminders, and self-management resources | Not applicable | Mixed asynchronous and interactive digital communication | ICU professionals, multidisciplinary team, peer survivors | Home/community | Activities: independence and daily activity; Participation: participation in meaningful daily, community, or social activities | Not explicitly reported |
| Kim et al [20], 2021 | ICU survivors (age ≥55 years) | Wearable monitoring | Continuous monitoring of physical activity, sedentary behavior, sleep, and heart rate to assess post-ICU frailty recovery | From hospital discharge to 4-week follow-up | Self-monitoring using wearable device | Self-directed with research team support | Home | Activities: daily step count, active time, Katz ADL | Not explicitly reported |
| Cha et al [21], 2026 | Non-ambulatory post-ICU patients (FAC ≤3) | Mobile health (mHealth) | Individualized app-guided upper- and lower-limb and trunk exercises with progressive strengthening | 2 weeks, 2 sessions/day | App-based video-guided exercise with initial in-person support | Rehabilitation physician and physiotherapist | General ward | Body functions and structures: MRC-SS, handgrip strength; Activities: ICU Mobility Scale, FAC | Not explicitly reported |
| Fortune et al [22], 2026 | ICU survivors with moderate-to-severe anemia | Wearable monitoring | Wrist-worn activity monitoring of free-living physical performance | 4 days at 1 and 3 months post-hospitalization | Wearable remote monitoring | Self-directed with research team support | Home/community | Activities: daily step count, maximum cadence, 6MWT | Not explicitly reported |
| Rosa et al [23], 2026 | Adults with acute hypoxemic respiratory failure requiring invasive ventilation | Telerehabilitation | ICU, ward, and postdischarge rehabilitation | ICU and ward phases, plus 2 months postdischarge | Telehealth-supported | Multidisciplinary team | ICU, ward, and home | Activities: new disability, physical dependence; Participation: return to work | Not explicitly reported |
| Lathrop et al [24], 2026 | Severe burn survivor requiring prolonged ICU care | Virtual reality (VR) | Immersive motor training combined with mobilization, strengthening, and ADL training | 4 weeks, 3 sessions/week, 60 min/session | Therapist-supervised immersive VR | Physiotherapist and occupational therapist | ICU/general rehabilitation ward | Activities: FIM, ambulation, functional independence | Not explicitly reported |
| Boehm et al [25], 2026 | ICU survivors with sepsis and/or ARDS | Telemedicine | Multidisciplinary virtual ICU recovery clinic with clinical review, medication management, psychological assessment, and recovery follow-up | Visits at 3 weeks and 3 months after discharge | Synchronous videoconferencing via secure web portal | ICU clinician, pharmacist, and psychologist | Home | Activities: ADL, IADL | Not explicitly reported |
| Ghiglieri et al [26], 2026 | ICU survivors requiring invasive MV ≥48 h | Telerehabilitation | Multicomponent rehabilitation including symptom management, psychological support, goal setting, individualized exercise/activity planning, group exercise, and peer support | 6 weeks | Synchronous video or telephone with optional recorded/live group exercise | iRehab intervention specialist | Home | Activities: qualitative experiences of exercise, activity, and physical recovery | Theoretical Framework of Acceptability (TFA); used to guide the qualitative study, interview approach, and analysis |
| Apps et al [27], 2026 | ICU survivors requiring MV ≥5 days | Digital recovery platform | Functional assessment, personalized goal setting, GAS monitoring, recovery resources, e-diary, reminders, and secure communication | 12 weeks | Mobile app with asynchronous content and text/audio/video communication | ICU recovery coordinator (occupational therapist) | Home | Activities: recovery goals related to mobility, daily activities, or functional independence; Participation: recovery goals explicitly related to meaningful life roles or participation. Each goal was assigned to the single ICF domain it most directly represented. | Canadian Model of Occupational Performance and Engagement (CMOP-E), used to structure the recovery goal menu; Theoretical Framework of Acceptability (TFA), used as an analytic lens |
| Note: Mobility-related outcomes were mapped to the International Classification of Functioning, Disability and Health domains of body functions and structures, activities, and participation. Rows represent the 27 included reports; linked reports arising from the same parent study were retained, with each parent study counted once among the 24 unique parent studies. Each individual mobility outcome or recovery goal was assigned to one ICF domain only, according to the domain it most directly represented, and was not double-counted across domains.  Abbreviations: 6MWT, 6-minute walk test; ACT, Arm Curl Test; ADL, activities of daily living; ARDS, acute respiratory distress syndrome; COVID-19, coronavirus disease 2019; FAC, Functional Ambulation Category; FIM, Functional Independence Measure; GAS, Goal Attainment Scaling; HRQoL, health-related quality of life; ICU, intensive care unit; IADL, instrumental activities of daily living; MDT, multidisciplinary team; mHealth, mobile health; MMI, Morton Mobility Index; MRC-SS, Medical Research Council Sum Score; MV, mechanical ventilation; SF-36 PCS, Physical Component Summary of the 36-Item Short Form Health Survey; SPPB, Short Physical Performance Battery; STST, sit-to-stand test; TUG, Timed Up and Go test; VR, virtual reality. | | | | | | | | | |

# Table S2. Statistical and analytical approaches and handling of Home-Generated digital and device Data

| **Study** | **Main statistical or analytical approach** | **Handling of home-generated digital and device data** |
| --- | --- | --- |
| de Vries et al [1], 2025 | Descriptive statistics; Wilcoxon signed-rank tests for paired pre–post comparisons | Not reported |
| Balakrishnan et al [2], 2023 | Descriptive statistics; χ², Fisher exact, *t*, and Wilcoxon rank-sum tests for between-group comparisons; content analysis of interview responses | Participants were instructed to obtain home vital-sign measurements at consistent times; no explicit artifact filtering, validity thresholds, or data-cleaning procedures were reported |
| Capin et al [3], 2022 | χ² and independent-sample *t* tests for baseline comparisons; covariate-adjusted mixed-effects models for longitudinal functional and patient-reported outcomes | Study staff verified Fitbit wear and synchronization during supervised sessions and weekly follow-up; no explicit minimum wear-time or signal-level filtering criteria were reported |
| Şahin et al [4], 2023 | Shapiro–Wilk testing; independent-sample *t* or Mann–Whitney U tests; two-way ANOVA with Bonferroni correction | Not reported |
| Khan et al [5], 2024 | Intention-to-treat mixed-effects models for longitudinal outcomes; χ² tests; time-to-event models and Andersen–Gill models for recurrent events; missing-data sensitivity analyses | Not reported |
| Trzmiel et al [6], 2023 | Student *t* or Mann–Whitney U tests; repeated-measures ANOVA with group-by-time effects; Greenhouse–Geisser correction when required; Tukey post hoc tests | Not reported |
| O’Neill et al [7], 2026 | Covariate-adjusted linear regression for the primary outcome; complier-average causal effect analyses; multiple-imputation sensitivity analyses; linear regression for continuous secondary outcomes | Not reported |
| Mayer et al [8], 2021 | Descriptive statistics; Welch independent *t* tests; paired *t* tests; exploratory equivalence testing | Not reported |
| Carpallo-Porcar et al [9], 2025 | Intention-to-treat parametric and nonparametric group/time comparisons; Friedman and Wilcoxon tests; categorical-data tests; Cohen *d* effect sizes; linear regression for associations with self-efficacy | Not reported |
| Howroyd et al [10], 2023 | Mann–Whitney U and χ² tests; Wilcoxon matched-pairs signed-rank tests for pre–post change | Not reported |
| Hunter et al [11], 2022 | Descriptive longitudinal analysis; Shapiro–Wilk testing; two-tailed Student *t* tests | A month was analyzed only when >10 days of smartwatch data were available; participants with no uploaded data were excluded; month 1 and month 12 data were required for 1-year comparative analyses |
| Ramalingam et al [12], 2020 | Descriptive case analysis; no formal inferential statistical model reported | Not reported |
| Rose et al [13], 2022 | Descriptive implementation and service-evaluation reporting; no formal inferential statistical model reported | Not reported |
| Taylor et al [14], 2022 | Intention-to-treat covariate-adjusted logistic regression; subgroup analyses; modified intention-to-treat and per-protocol sensitivity analyses | Not reported |
| Kovaleva et al [15], 2023 | Conventional qualitative content analysis with inductive coding and constant comparison | Not reported |
| Parker et al [16], 2020 | Descriptive statistics; inductive thematic analysis of semistructured interviews | Not reported |
| Vlake et al [17], 2022 | Mixed-effects linear and logistic regression models accounting for study site and repeated follow-up | Not reported |
| Martin et al [18], 2021 | Paired *t* or Wilcoxon tests; Spearman correlation; χ² tests | Not reported |
| Zacharelou et al [19], 2025 | Thematic analysis of focus-group data | Not reported |
| Kim et al [20], 2021 | Parametric/nonparametric univariable comparisons; Pearson and Spearman correlations; participant-level linear regression of longitudinal wearable-data trends | Participants with <5 days of wearable data were excluded from longitudinal trend analyses; technical failures resulting in data loss were documented |
| Cha et al [21], 2026 | Independent-sample t tests, χ² or Fisher exact tests, as appropriate; linear mixed-effects models with random intercepts; Bonferroni-adjusted pairwise comparisons; Friedman tests | Application usage logs were used to quantify exercise compliance; no explicit data-cleaning or validity criteria for app-generated data were reported |
| Fortune et al [22], 2026 | Spearman correlations; repeated-measures linear mixed-effects models; assessment of missing-data mechanism; complete-case analysis | ≥2 valid days with ≥10 h/day wear time were required; noncompliant observations were excluded; 60-second epochs and the ActiGraph default filter algorithm were used; outcomes were averaged across valid days |
| Rosa et al [23], 2026 | Generalized linear mixed model using the Hussey–Hughes approach; mixed-effects logistic and linear regression; Markov longitudinal ordinal model; multiple-imputation and zero-inflated Gaussian mixed-effects sensitivity analyses | Not reported |
| Lathrop et al [24], 2026 | Wilcoxon signed-rank tests for within-patient pain change; Spearman rank correlations | Not reported |
| Boehm et al [25], 2026 | Descriptive statistics; Mann–Whitney U tests; exploratory hierarchical linear growth modeling; assessment of missingness, outliers, and data integrity | Not reported |
| Ghiglieri et al [26], 2026 | Descriptive analysis of acceptability questionnaire scores; combined inductive–deductive Framework Method guided by the Theoretical Framework of Acceptability | Not reported |
| Apps et al [27], 2026 | Descriptive analysis of Likert-scale questionnaire responses; inductive reflexive thematic analysis using the Theoretical Framework of Acceptability as a flexible analytic lens | Not reported |
| Note: Statistical and analytical approaches are reported as described in the original studies. The handling of home-generated digital and device data refers to explicitly reported procedures addressing data acquisition, synchronization, completeness, validity, filtering, missingness, or technical failure. “Not reported” indicates that no explicit procedures for handling home-generated digital or device data were described in the original report; in some studies, such data were not collected. | | |

# Table S3. Primary and secondary clinical outcome findings in controlled and comparative studies

| **Study** | **Study comparison** | **Primary outcome** | **Primary between-group finding** | **Secondary mobility-related outcomes** | **Secondary between-group finding** | **Clinical-effectiveness interpretation** |
| --- | --- | --- | --- | --- | --- | --- |
| Balakrishnan et al [2], 2023 | Telemedicine clinic vs control; exploratory randomized controlled trial | Health care utilization within 30 days | Utilization was not significantly reduced. Time to first post-discharge visit was shorter with telemedicine (16.7 vs 29.4 days; P<.001). | EQ-5D mobility and usual-activities domains; 6-minute walk test completion | No significant differences in EQ-5D mobility or usual activities. The 6-minute walk test was assessed only in the intervention group. | No demonstrated between-group mobility benefit. Earlier follow-up reflects care delivery. |
| Capin et al [3], 2022 | App-facilitated telerehabilitation vs control; pilot randomized feasibility trial | Feasibility and safety; no clinical-effectiveness primary outcome | No primary clinical-effectiveness comparison was reported. | 30-second chair-stand test, Timed Up and Go, balance, and daily step count | No significant between-group differences in chair-stand change (P=.06), Timed Up and Go (P=.21), balance (P=.90), or daily step count. | The study supports feasibility; exploratory clinical comparisons did not demonstrate superiority. |
| Şahin et al [4], 2023 | Home pulmonary rehabilitation with telecoaching vs the same program without telecoaching; randomized controlled trial | 6-minute walk distance | Both groups improved; no significant between-group advantage in 6-minute walk distance was reported. | Muscle strength; activity-related dyspnea and exertional fatigue | Dyspnea and exertional fatigue favored telecoaching (P<.05). No clear between-group benefit in muscle strength was reported. | Comparative benefits were reported for symptoms; within-group improvement in walking distance does not show superiority. |
| Khan et al [5], 2024 | Mobile Critical Care Recovery Program vs telephone attention control; randomized controlled trial | 36-Item Short Form physical and mental component summary scores at 12 months | Adjusted differences in change: 1.61 (95% CI, −1.06 to 4.29; P=.24) for the physical component summary and −2.50 (95% CI, −5.29 to 0.30; P=.08) for the mental component summary; neither difference was significant. | Short Physical Performance Battery was planned but not completed. | No final performance-based mobility comparison was available. | No significant benefit was shown for either component of the primary quality-of-life outcome; mobility performance could not be assessed. |
| Trzmiel et al [6], 2023 | Robot-assisted plus neurological rehabilitation vs standard neurological rehabilitation; randomized controlled trial | The study did not specify a primary/secondary outcome hierarchy. | The group-by-time interaction was significant only for the Functional Independence Measure motor subscale (P=.047); post-treatment between-group comparisons were not significant. | Functional Independence Measure, Berg Balance Scale, Tinetti Scale, 6-minute walk test, Barthel Index, handgrip and elbow strength | No consistent between-group advantage across functional outcomes. | One significant interaction provides limited comparative evidence; improvement over time in both groups does not establish overall robot superiority. |
| O’Neill et al [7], 2026 | Remote multicomponent rehabilitation vs standard care; pragmatic randomized controlled trial | EQ-5D-5L utility score at 8 weeks | Adjusted mean difference 0.04 (95% CI −0.001 to 0.09; P=.05); the primary outcome was not significantly improved. | 30-second sit-to-stand test | Adjusted mean difference 1.0 repetition (95% CI 0.3–1.6; P=.01), favoring the intervention. | The prespecified primary HRQoL outcome at 8 weeks was not significantly improved, whereas the secondary sit-to-stand outcome favored the intervention. |
| Mayer et al [8], 2021 | Telehealth vs in-person recovery pathways; nonrandomized study with self-selected pathways | Safety and feasibility | No clinical-effectiveness primary outcome was specified. | 6-minute walk distance, Short Physical Performance Battery, Timed Up and Go, Medical Research Council sum score, handgrip strength, and 2-minute step test | Post-assessment 6-minute walk distance did not differ significantly (P=.19). Equivalence was not established. | No clear between-pathway superiority or equivalence was demonstrated. |
| Carpallo-Porcar et al [9], 2025 | App/platform-based rehabilitation vs booklet-based rehabilitation; secondary analysis of a pilot randomized trial | The report focused on self-efficacy and physical-activity motivation; no mobility-effectiveness primary outcome was specified. | Self-efficacy favored the app group at 3 and 6 months (P=.025 and P=.007). | 6-minute walk test, 30-second sit-to-stand, Arm Curl Test, and post-COVID functional scale | No significant immediate between-group mobility differences were reported. Correlations do not estimate intervention effects. | The findings concern self-efficacy; mobility superiority was not demonstrated. This is a secondary report from the parent trial. |
| Hunter et al [11], 2022 | Multidisciplinary-team review of smartwatch data vs usual follow-up at control sites; observational comparison | No prespecified hierarchy; step count was the principal mobility measure. | Step count increased more at the multidisciplinary-team site than at control sites (176% vs 42%; +5,474 vs +2,181 steps; P=.04). | No other secondary mobility outcome was clearly specified. | Not reported separately. | The site-level observational association does not establish a causal effect; site differences and missing data limit interpretation. |
| Taylor et al [14], 2022 | Sepsis Transition and Recovery (STAR) program vs usual care; multisite pragmatic randomized clinical trial | Composite of all-cause mortality or hospital readmission within 30 days after discharge | Primary outcome occurred in 28.7% vs 33.3%; adjusted OR 0.80 (95% CI, 0.64–0.98) | No mobility-specific secondary outcome was reported | No mobility-specific between-group effect was assessed. Outpatient rehabilitation/physical or occupational therapy during follow-up was similar between groups (17% vs 16%; P=.99) as a care-process measure | The adjusted primary analysis favored STAR for mortality/readmission; no mobility-specific clinical effectiveness was evaluated. |
| Vlake et al [17], 2022 | ICU-specific virtual reality plus usual follow-up vs usual follow-up; randomized controlled trial | Psychological distress and health-related quality of life through 6 months | No consistent between-group benefit was reported across psychological or quality-of-life outcomes. | Physical quality-of-life domains. No performance-based mobility test was reported. | No mobility-specific between-group benefit was reported. Higher aftercare satisfaction is an acceptability outcome. | The study did not demonstrate a mobility benefit. Satisfaction should not be interpreted as clinical improvement. |
| Martin et al [18], 2021 | Voluntary telerehabilitation vs nonparticipating controls; observational comparison | No prespecified hierarchy; 1-minute sit-to-stand was the principal functional measure. | Sit-to-stand improvement was greater with telerehabilitation (P=.005). | Dyspnea and physiological responses during sit-to-stand | Dyspnea change was similar between groups. | The comparative result is exploratory; voluntary participation and nonrandomization limit causal inference. |
| Rosa et al [23], 2026 | Integrated telehealth rehabilitation vs usual care (stepped-wedge cluster randomized trial) | EQ-5D-3L utility at 90 days after discharge | Higher in the intervention group: adjusted difference 0.049 (95% CI, 0.0002–0.098; P = .04). Among survivors, no significant difference (P = .34). | Activities: new disability, physical dependence; Participation: return to work | No significant between-group differences | Higher overall utility was accompanied by lower mortality; no benefit was found for the reported mobility-related outcomes. |
| Boehm et al [25], 2026 | Multidisciplinary telemedicine ICU recovery clinic vs standard care; two-site pilot randomized trial | Feasibility of trial procedures and intervention delivery; no clinical primary outcome | No confirmatory clinical-effectiveness comparison was planned. | Exploratory physical-health composite, including PROMIS Physical Function | No significant between-group change was reported in physical-health or other post-intensive-care syndrome composite scores. | The study was designed for feasibility and was not powered to test clinical effectiveness; exploratory results do not establish benefit or equivalence. |
| Abbreviations: CI, confidence interval; EQ-5D-3L, EuroQol 5-Dimension 3-Level; EQ-5D-5L, EuroQol 5-Dimension 5-Level; ICU, intensive care unit; MDT, multidisciplinary team; PROMIS, Patient-Reported Outcomes Measurement Information System; RCT, randomized controlled trial; SD, standard deviation. | | | | | | |

# Table S4. Feasibility, adherence, safety, and implementation findings of digital health interventions

| **Author, year** | **Feasibility/implementation domain** | **Attrition** | **Adherence or implementation performance** | **Reasons for non-completion** | **Key feasibility/implementation finding** |
| --- | --- | --- | --- | --- | --- |
| de Vries et al [1], 2025 | Satisfaction, session efficiency, adherence | 1/10 did not complete training | 60% of planned VR sessions completed; session efficiency 25%–93% | Fatigue; inability to sit adequately | VR therapy was feasible during ICU and ward admission |
| Balakrishnan et al [2], 2023 | Self-monitoring completion | Not specifically reported | Daily vital-sign logs: 44% complete, 33% partial; 6MWT completed by 50% | Lack of time, lost monitoring sheets, smartphone malfunction | Self-monitoring completion was moderate; no adverse events occurred |
| Capin et al [3], 2022 | Safety and session adherence | Not clearly reported | 27/29 (93%) attended ≥75% of supervised sessions | Perceived no benefit, loss of interest, loss to follow-up | Telerehabilitation was feasible, safe, and had high adherence |
| Şahin et al [4], 2023 | Exercise adherence and safety | Not reported | Compliance: 90% with telecoaching vs 70% in control | Not reported | Telecoaching was associated with higher programme compliance; no side effects were reported |
| O’Neill et al [7], 2026 | Adherence and intervention fidelity | 4/231 withdrew; 6/231 discontinued intervention | Full adherence 73.2%; ≥1 session 88.7%; no adherence 11.2%; fidelity remained high | Not contactable, discontinuation, withdrawal | Remote rehabilitation achieved substantial engagement with high delivery fidelity |
| Mayer et al [8], 2021 | Recruitment, retention, adherence, safety | 3/32 (9.4%) | Clinic attendance 88%; physical therapy 78.4%; home exercise 65%; walking programme 62% | Medical complications/readmission; personal reasons | The programme was feasible; directly related adverse events occurred in 4/325 sessions (1.2%) |
| Howroyd et al [10], 2023 | Recruitment, retention, adherence | 10/38 did not complete | Retention 73.6%; adherence 82% | Technology access; language barriers | Programme delivery was feasible with good adherence |
| Hunter et al [11], 2022 | Wearable use and data capture | 15/50 lacked usable uploaded data | 35/50 provided usable smartwatch data | Not reported | Wearable monitoring was feasible, although missing data were substantial |
| Taylor et al [14], 2022 | Navigator engagement and care-process implementation | Not reported | Median 15 navigator contacts; follow-up within 10 days 39% vs 31%; medication reconciliation 76% vs 50% | Not reported | STAR increased delivery of several recommended postsepsis care processes |
| Parker et al [16], 2020 | Prototype usability and task completion | 0/10 dyads after enrolment | 10/10 dyads completed study procedures | Not reported | The app prototype was feasible to use in the home setting |
| Vlake et al [17], 2022 | VR session completion and feasibility | Not clearly reported | Intervention participants completed the single ICU-VR session | Not reported | ICU-VR was feasible to deliver without reported safety concerns |
| Martin et al [18], 2021 | Programme completion and safety | 1/15 withdrew before final evaluation | 14/15 (93.3%) completed the 6-week programme | Privacy concerns | Telerehabilitation was feasible and safe; no adverse events occurred |
| Kim et al [20], 2021 | Wearable data collection | 4/16 incomplete data | 12/16 (75%) provided usable data; mean wear duration 26.3 days | Withdrawal; technical failures | Wearable monitoring was feasible, although incomplete data and technical failures occurred |
| Cha et al [21], 2026 | Retention, exercise adherence, safety | 5/25 did not complete the study; retention was 80% (20/25). | Retention 80%; mean exercise compliance 40.6% | Clinical deterioration or transfer | The app-based programme was feasible and safe, although adherence varied |
| Fortune et al [22], 2026 | Completion of wearable monitoring | Not reported | >80% completed monitoring and provided valid data | Not reported | Home activity monitoring was feasible after critical illness |
| Rosa et al [23], 2026 | Intervention fidelity and participation | 229/1,916 not included in primary analysis | Overall fidelity 69.6%; ICU 80.4%; ward 65.0%; postdischarge 61.8% | Transfer; loss to follow-up | Implementation was feasible across 20 hospitals, with lower participation after discharge |
| Lathrop et al [24], 2026 | Completion of VR rehabilitation | Not reported | All 12 planned sessions completed | Not reported | The VR rehabilitation protocol was feasible to deliver in this case |
| Boehm et al [25], 2026 | Recruitment, retention, visit attendance, fidelity | 29/91 did not complete 6-month follow-up | 23/40 eligible intervention participants attended ≥1 visit | Death, withdrawal, loss to follow-up | Telemedicine delivery was feasible, although attendance and retention were challenges |
| Apps et al [27], 2026 | Perceived feasibility of the digital pathway | Not reported | ≥80% agreed with 3/4 feasibility items among 59 questionnaire respondents | Not reported | The digital recovery pathway was generally perceived as feasible |
| Abbreviations: 6MWT, 6-minute walk test; ARF, acute respiratory failure; ICU, intensive care unit; MDT, multidisciplinary team; STAR, Sepsis Transition and Recovery; VR, virtual reality. Note: Only studies reporting feasibility, adherence, safety, fidelity, retention, intervention completion, or other implementation outcomes were included. Outcomes are reported as defined in the original studies. Percentages refer to the intervention group unless otherwise specified. Implementation findings were synthesized separately from clinical-effectiveness outcomes. “Not reported” indicates that the information was unavailable in the original report. | | | | | |
|  | | | | | |

# Table S5. Acceptability outcomes and findings of digital health interventions

| **Author, year** | **Acceptability/usability measure** | **Main finding** | **Facilitators** | **Barriers** |
| --- | --- | --- | --- | --- |
| de Vries et al [1], 2025 | VAS satisfaction score; patient/trainer feedback | High satisfaction; median 80/100 | Enjoyment, immersion, motivation | Fatigue, weakness, headset support, technical interruptions |
| O’Neill et al [7], 2026 | Theoretical Framework of Acceptability Questionnaire (TFAQ) | Acceptability favored remote rehabilitation (adjusted mean difference 0.8, 95% CI 0.6–1.0; P<.001) | Explored in linked Ghiglieri 2026 qualitative study | Explored in linked Ghiglieri 2026 qualitative study |
| Hunter et al [11], 2022 | Patient and MDT feedback questionnaires | Participants reported that Fitbit use motivated their recovery (71% at the MDT site vs 48% at control sites); 86% vs 48% reported aiming to increase their activity. | Real-time activity feedback; MDT review | Device/app compatibility; incomplete data upload |
| Kovaleva et al [15], 2023 | Qualitative interviews with patients and caregivers | Telemedicine follow-up was viewed as acceptable, convenient, and reassuring | Convenience, reduced travel, multidisciplinary input, caregiver participation | Scheduling, insufficient individualization, technical ability |
| Parker et al [16], 2020 | SUS and semistructured interviews | SUS: patients 76 [68–83], care partners 88 [75–94]; app was usable and acceptable | Recovery motivation, goal tracking, multidisciplinary support | Depression-related stigma; device and accessibility concerns |
| Vlake et al [17], 2022 | Satisfaction and aftercare-rating questionnaires | ICU aftercare satisfaction was higher with ICU-VR (8.7 vs 7.6; P=.006); aftercare rating was also higher (8.9 vs 7.8; P=.002) | Informational content and improved understanding of ICU treatment | Not reported |
| Zacharelou et al [19], 2025 | Qualitative focus group interviews | Survivors valued personalized, accessible digital follow-up with reliable information and communication | Tailored information, e-consultation, peer support, digital ICU diary | Lack of personalization; unclear support pathways; accessibility needs |
| Cha et al [21], 2026 | Satisfaction and app-experience questionnaire | Satisfaction was 4.4/5 at week 1 and 4.3/5 at week 2; ease of use was 4.3/5 and 4.6/5 | Ease of use and perceived exercise benefit | Not reported |
| Lathrop et al [24], 2026 | User Satisfaction Evaluation Questionnaire; session ratings | Overall satisfaction was 93.33%; median session satisfaction 6 [5–7] | Not clearly reported | Not reported |
| Boehm et al [25], 2026 | Acceptability of Intervention Measure | Median acceptability was 4.13 [4–5] among 16 telemedicine participants | Not reported | Not reported |
| Ghiglieri et al [26], 2026 | TFA-guided interviews and modified TFAQ | Remote rehabilitation was generally acceptable, although experiences varied | Structure, continuity, confidence, individualized support, optional peer support | Digital burden; differing readiness, circumstances, and capability |
| Apps et al [27], 2026 | Acceptability of Intervention Measure and semistructured interviews | ≥80% of 59 respondents agreed with all 4 acceptability items | Personalized goals, motivation, accountability, reassurance, coordinator support | Technical complexity, notification burden, physical/cognitive challenges |
| Abbreviations: AIM, Acceptability of Intervention Measure; IQR, interquartile range; MDT, multidisciplinary team; SUS, System Usability Scale; TFA, Theoretical Framework of Acceptability; TFAQ, Theoretical Framework of Acceptability Questionnaire; VAS, visual analogue scale; VR, virtual reality. Note: Only studies that directly assessed acceptability, usability, satisfaction, or user experience were included. Facilitators and barriers were reported only when explicitly identified by participants, providers, or the original study authors. “Not reported” indicates that the corresponding information was not provided in the original report. Linked reports from the same parent study were retained when they provided distinct acceptability evidence. Acceptability, usability, satisfaction, and user-experience findings were interpreted separately from evidence of clinical effectiveness. | | | | |

References

1. de Vries M, Beumeler LFE, van der Meulen J, Bethlehem C, den Otter R, Boerma EC. The feasibility of virtual reality therapy for upper extremity mobilization during and after intensive care unit admission. PeerJ. 2025;13:e18461. [doi: 10.7717/peerj.18461] [Medline: 39802193]

2. Balakrishnan B, Hamrick L, Alam A, Thompson J. Effects of COVID-19 acute respiratory distress syndrome intensive care unit survivor telemedicine clinic on patient readmission, pain perception, and self-assessed health scores: randomized, prospective, single-center, exploratory study. JMIR Form Res. Mar 22, 2023;7:e43759. [doi: 10.2196/43759] [Medline: 36877802]

3. Capin JJ, Jolley SE, Morrow M, et al. Safety, feasibility and initial efficacy of an app-facilitated telerehabilitation (AFTER) programme for COVID-19 survivors: a pilot randomised study. BMJ Open. Jul 26, 2022;12(7):e061285. [doi: 10.1136/bmjopen-2022-061285] [Medline: 35882451]

4. Şahin H, Naz İ, Karadeniz G, Süneçli O, Polat G, Ediboğlu O. Effects of a home-based pulmonary rehabilitation program with and without telecoaching on health-related outcomes in COVID-19 survivors: a randomized controlled clinical study. J Bras Pneumol. 2023;49(1):e20220107. [doi: 10.36416/1806-3756/e20220107] [Medline: 36700571]

5. Khan BA, Perkins AJ, Khan SH, et al. Mobile critical care recovery program for survivors of acute respiratory failure: a randomized clinical trial. JAMA Netw Open. Jan 2, 2024;7(1):e2353158. [doi: 10.1001/jamanetworkopen.2023.53158] [Medline: 38289602]

6. Trzmiel T, Marchewka R, Pieczyńska A, et al. The effect of using a rehabilitation robot for patients with post-coronavirus disease (COVID-19) fatigue syndrome. Sensors (Basel). Sep 27, 2023;23(19):8120. [doi: 10.3390/s23198120] [Medline: 37836950]

7. O’Neill B, Bradley JM, Connolly B, et al. Remote multicomponent rehabilitation in intensive care unit survivors: a randomized clinical trial. JAMA. Jul 21, 2026;336(3):236-247. [doi: 10.1001/jama.2026.7401] [Medline: 42150121]

8. Mayer KP, Parry SM, Kalema AG, et al. Safety and feasibility of an interdisciplinary treatment approach to optimize recovery from critical coronavirus disease 2019. Crit Care Explor. Aug 2021;3(8):e0516. [doi: 10.1097/CCE.0000000000000516] [Medline: 34476403]

9. Carpallo-Porcar B, Jiménez-Sánchez C, Liñares-Varela I, et al. Impact of an asynchronous telerehabilitation program on the self-efficacy and motivation for physical activity in discharged COVID-19 patients: a secondary analysis of a randomized controlled trial. Ann Med. Dec 2025;57(1):2573148. [doi: 10.1080/07853890.2025.2573148] [Medline: 41137860]

10. Howroyd F, Earle N, Weblin J, et al. Virtual post-intensive-care rehabilitation for survivors of COVID-19: a service evaluation. Cureus. May 2023;15(5):e38473. [doi: 10.7759/cureus.38473] [Medline: 37273405]

11. Hunter A, Leckie T, Coe O, et al. Using smartwatches to observe changes in activity during recovery from critical illness following COVID-19 critical care admission: 1-year, multicenter observational study. JMIR Rehabil Assist Technol. May 2, 2022;9(2):e25494. [doi: 10.2196/25494] [Medline: 35417402]

12. Ramalingam MB, Huang Y, Lim PAC. Rehabilitation of a post-intensive care unit patient after severe COVID-19 pneumonia. Am J Phys Med Rehabil. Dec 2020;99(12):1092-1095. [doi: 10.1097/PHM.0000000000001606] [Medline: 33002914]

13. Rose L, Apps C, Brooks K, Terblanche E, Hart N, Meyer J. Novel digitally enabled care pathway to support postintensive care recovery and goal attainment following critical illness. BMJ Innov. Jan 2022;8(1):42-47. [doi: 10.1136/bmjinnov-2021-000842]

14. Taylor SP, Murphy S, Rios A, et al. Effect of a multicomponent sepsis transition and recovery program on mortality and readmissions after sepsis: the Improving Morbidity During Post-Acute Care Transitions for Sepsis randomized clinical trial. Crit Care Med. Mar 1, 2022;50(3):469-479. [doi: 10.1097/CCM.0000000000005300] [Medline: 34534130]

15. Kovaleva MA, Jones AC, Kimpel CC, et al. Patient and caregiver experiences with a telemedicine intensive care unit recovery clinic. Heart Lung. 2023;58:47-53. [doi: 10.1016/j.hrtlng.2022.11.002] [Medline: 36399862]

16. Parker AM, Nelliot A, Chessare CM, et al. Usability and acceptability of a mobile application prototype for a combined behavioural activation and physical rehabilitation intervention in acute respiratory failure survivors. Aust Crit Care. Nov 2020;33(6):511-517. [doi: 10.1016/j.aucc.2020.02.010] [Medline: 32340769]

17. Vlake JH, van Bommel J, Wils EJ, et al. Intensive care unit-specific virtual reality for critically ill patients with COVID-19: multicenter randomized controlled trial. J Med Internet Res. Jan 31, 2022;24(1):e32368. [doi: 10.2196/32368] [Medline: 34978530]

18. Martin I, Braem F, Baudet L, et al. Follow-up of functional exercise capacity in patients with COVID-19: It is improved by telerehabilitation. Respir Med. Jul 2021;183:106438. [doi: 10.1016/j.rmed.2021.106438] [Medline: 33964817]

19. Zacharelou A, Munjas Samarin R, Mikulcic K, et al. Value-based experiences related to digital follow-up services among critical care survivors: an international qualitative study. Nurs Health Sci. Jun 2025;27(2):e70135. [doi: 10.1111/nhs.70135] [Medline: 40390388]

20. Kim B, Hunt M, Muscedere J, Maslove DM, Lee J. Using consumer-grade physical activity trackers to measure frailty transitions in older critical care survivors: exploratory observational study. JMIR Aging. Feb 23, 2021;4(1):e19859. [doi: 10.2196/19859] [Medline: 33620323]

21. Cha S, Kim YJ, Lee C, et al. The feasibility, safety, and preliminary functional outcomes of a mobile application-based rehabilitation program in non-ambulatory patients after intensive care unit discharge. J Clin Med. May 29, 2026;15(11):4211. [doi: 10.3390/jcm15114211] [Medline: 42279075]

22. Fortune E, Hanson AC, Faust L, et al. Feasibility of activity monitors to assess physical capacity after critical illness: an observational study nested within a randomized clinical trial. Mayo Clin Proc Digit Health. 2026;4(3):100388. [doi: 10.1016/j.mcpdig.2026.100388] [Medline: 42603965]

23. Rosa RG, Moraes RB, Trott G, et al. Integrated telehealth rehabilitation and quality of life in mechanically ventilated adults: a randomized clinical trial. JAMA. Sep 15, 2026;336(11):939-949. [doi: 10.1001/jama.2026.10617] [Medline: 42268591]

24. Lathrop C, Pino C, Peralta-Wieland B. Functional immersive rehabilitation for a severely burned patient with refractory pain: a case report. Physiother Theory Pract. Aug 2026;42(8):1112-1119. [doi: 10.1080/09593985.2026.2615378] [Medline: 41524267]

25. Boehm LM, You H, Siao SF, et al. Multidisciplinary telemedicine intervention for ICU recovery: the TelePORT feasibility randomized trial. Crit Care. Jun 27, 2026;30(1):476. [doi: 10.1186/s13054-026-06154-y] [Medline: 42365340]

26. Ghiglieri CG, Connolly BA, Murphy D, et al. Factors influencing the acceptability of remote multicomponent rehabilitation versus standard care following critical illness: a theory-informed qualitative study embedded within the iRehab trial. J Intensive Care. Jul 26, 2026;14(1):81. [doi: 10.1186/s40560-026-00908-0] [Medline: 42642751]

27. Apps C, Brooks K, Corcoran E, et al. Exploring the acceptability and feasibility of a digital intensive care unit recovery pathway with an embedded goal menu: a multiple-methods approach. Aust Crit Care. Aug 2026;39(4):101619. [doi: 10.1016/j.aucc.2026.101619] [Medline: 42275764]
